# The Mnn2 Mannosyltransferase Family Modulates Mannoprotein Fibril Length, Immune Recognition and Virulence of *Candida albicans*

**DOI:** 10.1371/journal.ppat.1003276

**Published:** 2013-04-25

**Authors:** Rebecca A. Hall, Steven Bates, Megan D. Lenardon, Donna M. MacCallum, Jeanette Wagener, Douglas W. Lowman, Michael D. Kruppa, David L. Williams, Frank C. Odds, Alistair J. P. Brown, Neil A. R. Gow

**Affiliations:** 1 Aberdeen Fungal Group, Institute of Medical Sciences, University of Aberdeen, Foresterhill, Aberdeen, United Kingdom; 2 Biosciences, College of Life and Environmental Sciences, University of Exeter, Exeter, United Kingdom; 3 Quillen College of Medicine, East Tennessee State University, Johnson City, Tennessee, United States of America; 4 AppRidge International, LLC, Telford, Tennessee, United States of America; Albert Einstein College of Medicine, United States of America

## Abstract

The fungal cell wall is the first point of interaction between an invading fungal pathogen and the host immune system. The outer layer of the cell wall is comprised of GPI anchored proteins, which are post-translationally modified by both *N*- and *O*-linked glycans. These glycans are important pathogen associated molecular patterns (PAMPs) recognised by the innate immune system. Glycan synthesis is mediated by a series of glycosyl transferases, located in the endoplasmic reticulum and Golgi apparatus. Mnn2 is responsible for the addition of the initial α1,2-mannose residue onto the α1,6-mannose backbone, forming the *N*-mannan outer chain branches. In *Candida albicans*, the *MNN2* gene family is comprised of six members (*MNN2*, *MNN21*, *MNN22*, *MNN23*, *MNN24* and *MNN26*). Using a series of single, double, triple, quintuple and sextuple mutants, we show, for the first time, that addition of α1,2-mannose is required for stabilisation of the α1,6-mannose backbone and hence regulates mannan fibril length. Sequential deletion of members of the *MNN2* gene family resulted in the synthesis of lower molecular weight, less complex and more uniform *N*-glycans, with the sextuple mutant displaying only un-substituted α1,6-mannose. TEM images confirmed that the sextuple mutant was completely devoid of the outer mannan fibril layer, while deletion of two *MNN2* orthologues resulted in short mannan fibrils. These changes in cell wall architecture correlated with decreased proinflammatory cytokine induction from monocytes and a decrease in fungal virulence in two animal models. Therefore, α1,2-mannose of *N*-mannan is important for both immune recognition and virulence of *C. albicans*.

## Author Summary

*Candida albicans* is an opportunistic fungal pathogen of humans, which can cause superficial infections of the oral and genital epithelium or fatal systemic disease in immune compromised individuals. The cell wall of *C. albicans* consists of an inner layer of chitin and β-glucan and an outer fibrillar layer of highly glycosylated mannoproteins. These mannose-rich carbohydrates play crucial roles in fungal immune recognition. Deletion of genes encoding the Mnn2 family of α1,2-mannosyltransferases, which are involved in the biosynthesis of *N*-mannan outer chains, demonstrated that the addition of mannan side chains is necessary for the stabilisation and extension of the *N*-mannan backbone. Mutants of the *MNN2* gene family had marked alterations in mannan microfibril length. Reductions in the degree of *N*-mannan branching resulted in decreased immune recognition by monocytes, whilst complete removal of the branched outer chains increased β-glucan exposure, and correlated with an attenuated virulence. Therefore, the Mnn2 mannosyltransferase family is critical for microfibril structure, immune recognition and virulence.

## Introduction

*Candida albicans* is a polymorphic fungus that forms part of the natural human microflora. However, many adverse conditions result in predisposition to oral and vaginal infections and, under circumstances where the host immune system becomes severely compromised as a consequence of malignancy, trauma or chemotherapy, *C. albicans* can invade underlying epithelial cells and disseminate via the bloodstream and cause systemic disease. The associated mortality rate of systemic disease is approximately 30–40%, which is higher than that observed for many bacterial systemic infections, making *C. albicans* a major pathogen of the immunocompromised and a significant global health burden [1]–[3].

The fungal cell wall is a highly dynamic structural organelle essential for maintaining cell shape and for protection against the environment. The cell wall is also the first point of contact between the fungus and the host, and as a result the cell wall is pivotal for fungus-host interactions and immune recognition. The cell wall is comprised of an inner skeletal layer of chitin and β-glucans (β1,3- and β1,6-glucan) and an outer layer of highly glycosylated mannoproteins [4]–[6]. These proteins are decorated with linear *O*-linked mannan and highly branched *N*-linked mannan, which can be elaborated with additional mannan side chains attached via a phosphodiester linkage known as phosphomannan (PM). The mannan fraction of the cell wall is important for adhesion, cell wall integrity, immune recognition and comprises up to 40% of the cell wall dry weight [7]–[12]. All of the major cell wall carbohydrate components of fungal walls serve as pathogen associated molecular patterns (PAMPs), which are recognised by the innate immune system through pattern recognition receptors (PRRs) on the surface of immune effector cells [5], [13]–[17]. Mannans, which are exposed on the outer surface of the cell wall, are recognised by a wide range of PRRs depending on the immune cell type. These include toll-like receptor 4 (TLR4), TLR2, and the C-type lectins macrophage mannose receptor (MMR), MINCLE/CLEC4E, Dectin-2, galectin 3, and DC-SIGN/CD209 [16], [18]. β-Glucan, which is in the inner sub-mannan wall layer, but is exposed at bud scars, is recognised by the lectin-like receptor, Dectin-1 [13], [19]. More recently, chitin has been shown to participate in the immune recognition of *C. albicans*, possibly by damping the immune response during the resolution of infections [20], but the receptor(s) mediating this interaction are unknown.

Pioneering studies in *Saccharomyces cerevisiae* identified a series of mannosyltransferases, which are involved in mannan biosynthesis, and many of these enzymes are conserved in *C. albicans* and other pathogenic fungi. However, mannan structure differs significantly between fungal species, and this has important consequences for host-fungus interactions, and in the future development of vaccines and diagnostics. For example, *S. cerevisiae O*-mannan is comprised of two or more α1,2-mannose units, which are capped by several α1,3-mannose units [21]–[23], while the *O*-mannan in *C. albicans* is comprised solely of α1,2-mannose [11], [24]–[25]. In addition, the *N*-mannan extracted from *C. albicans* has a higher molecular weight than that extracted from *S. cerevisiae*, includes β1,2-mannan in both the acid-labile and acid-stable fractions as well as higher levels of phosphomannan [26]. Therefore, studies of mannan biosynthesis in *C. albicans* provide important and specific insights into the host interactions and immune recognition of this fungus.

Many mannosyltransferases are fungal-specific and are encoded by gene families. This complicates investigations of mannan biosynthesis due to possible functional redundancy between family members. In addition, families of mannosyltransferases can catalyse multiple reactions [9] whose structure-function relationships cannot yet be determined from their amino acid sequences or by structural proteomics. Progress in this field therefore requires a careful, detailed, simultaneous dissection of the function of entire gene families.

As proteins transverse though the endoplasmic reticulum (ER) the *N*-mannan precursor (Glc_3_Man_9_GlcNAc_2_) is attached to specific asparagine residues of the protein. This core is subsequently processed in the ER by glycosidases resulting in the mature *N*-mannan core (Man_8_GlcNAc_2_) [10]. Heavily branched outer chains are subsequently attached to the core as the protein passes through the Golgi. Initially, a single α1,6-mannose unit is attached to the core by Och1 [7]. This single mannose reside is then elaborated with α1,6-mannose residues, added by mannan polymerase I and II predominately composed of Mnn9, and form the α1,6-backbone [27]. The α1,6-backbone is further elaborated with side chains consisting of α1,2-, and α1,3-mannose residues, with the α1,2-mannose extending out from the backbone. In *S. cerevisiae*, the initial α1,2-mannose unit is attached to the backbone by mannosyltransferase, Mnn2, which is then elaborated with additional α1,2-mannose units by Mnn5 [28]. In *C. albicans*, these outer chains are then capped with either β1,2-mannose or α1,3-mannose units by Bmt1, Bmt3 and Mnn1 [29]–[30]. Deletion of *ScMNN2* inhibits the addition of α1,2-mannose onto the mannan backbone, thus preventing the elaboration of *N*-mannan outer chains [28]. In *C. albicans*, complete removal of the *N*-mannan outer chains has significant consequences for growth, cell morphology and immune recognition [7], although it is not known which of the deleted carbohydrate epitopes in the *och1*Δ mutant account for the lack of immune recognition.

Here, we functionally dissect the six-member *MNN2* gene family in *C. albicans*. To fully deduce the function(s) of each glycosyltransferase in *N*-mannan biosynthesis, we characterised a set of single, double, triple, quintuple and sextuple mutants. Deletion of even single *MNN2* gene family members reduced cell wall integrity and immune recognition. These effects were enhanced when multiple family members were deleted, with the sextuple mutant showing severe defects in cell wall integrity, virulence and immune recognition. We show that mannoprotein fibril length was progressively shortened in multiple *mnn2* family mutant backgrounds, that Mnn2 and Mnn26 are required for the addition of the initial α1,2-mannose residue to the α1,6-mannose backbone and that Mnn21, Mnn22, Mnn23 and Mnn24 are required for the addition of subsequent α1,2-mannose units onto the α1,6- α1,2-mannose scaffold.

## Results

### The six-member *C. albicans* Mnn2 mannosyltransferase family

To identify orthologues of *S. cerevisiae* Mnn2 in *C. albicans*, a Blastp search [31] of ScMnn2 was performed against the *C. albicans* genome in the *Candida* Genome Database (www.candidagenome.org). Six orthologues were identified (orf19.2347, orf19.1011, orf19.3803, orf19.4874, orf19.1995, orf19.6692) and designated *MNN2, MNN21, MNN22, MNN23, MNN24* and *MNN26*. A multiple sequence alignment for the six putative Mnn2 orthologues was generated with ClustalW2 (version 2.0.12) and from this alignment a phylogram for the *C. albicans MNN2* gene family was created (Figure S1). This phylogram identified three sub-groups: group 1 comprised of *MNN2, MNN22* and *MNN23*, group 2 comprised of *MNN21* and group 3 comprised of *MNN24* and *MNN26*. Initially each gene in the family was deleted individually and then, to test for possible functional redundancy, a series of multiple gene-disrupted mutants was constructed using the mini ura blaster protocol. This series was comprised of a double mutant for group 3 members (*mnn24*Δ/*mnn26*Δ), double mutants within groups 1 and 3 (*mnn2*Δ/*mnn26*Δ and *mnn23*Δ/*mnn26*Δ), a triple mutant for group 1 (*mnn2*Δ/*mnn22*Δ/*mnn23*Δ), a quintuple mutant for groups 1 and 3 (*mnn2*Δ/*mnn22*Δ/*mnn23*Δ*/mnn24*Δ/*mnn26*Δ) and the sextuple mutant (*mnn2*Δ/*mnn22*Δ/*mnn23*Δ*/mnn24*Δ/*mnn26*Δ/*mnn21*Δ). The multiple mutants were exposed to several rounds of 5-FOA treatment. Therefore, multiple colonies of each strain were tested for chromosomal loss or aneuploidy by a PCR based method [32]–[33]. No significant differences in the relative intensities of the PCR fragments were obtained in the strains used in functional analyses (data not shown), and no detectable changes were found in the karyotypes of constructs that had been repeatedly exposed to 5-FOA.

### Deletion of *MNN2* gene family members affects cell separation

All single mutants had growth rates similar to the parental control strain (doubling time 2.3±0.1 h) in YPD at 30°C, and the double, triple and quintuple mutants had slightly slower growth rates (doubling time 2.8±0.1 h). The sextuple mutant showed the slowest growth (doubling time 3.1±0.3 h). All mutants except *mnn21*Δ, *mnn22*Δ and *mnn24*Δ showed an increase in aggregation when grown in YPD at 30°C, suggesting a cell separation defect, as reported for other mannosylation mutants [7], [9]–[11]. Reintegration of a single copy of the respective gene into the *RPS1* locus was sufficient to restore wild type morphology in the six single mutants, but reintegration of *MNN2* and *MNN26* at the *RPS1* locus was not sufficient to completely prevent cell aggregation in the sextuple mutant (data not shown). To deduce whether the mannosylation defects affected morphogenesis the mutants were plated on Spider medium, DMEM supplemented with 5% serum and DMEM supplemented with 5% CO_2_. All strains produced filaments on the DMEM media, but the *mnn22*Δ, the quintuple and the sextuple mutants showed delayed filamentation on Spider medium (data not shown).

### The *MNN2* gene family provides the mannan scaffold to which phosphomannan is attached

**Figure 1:**
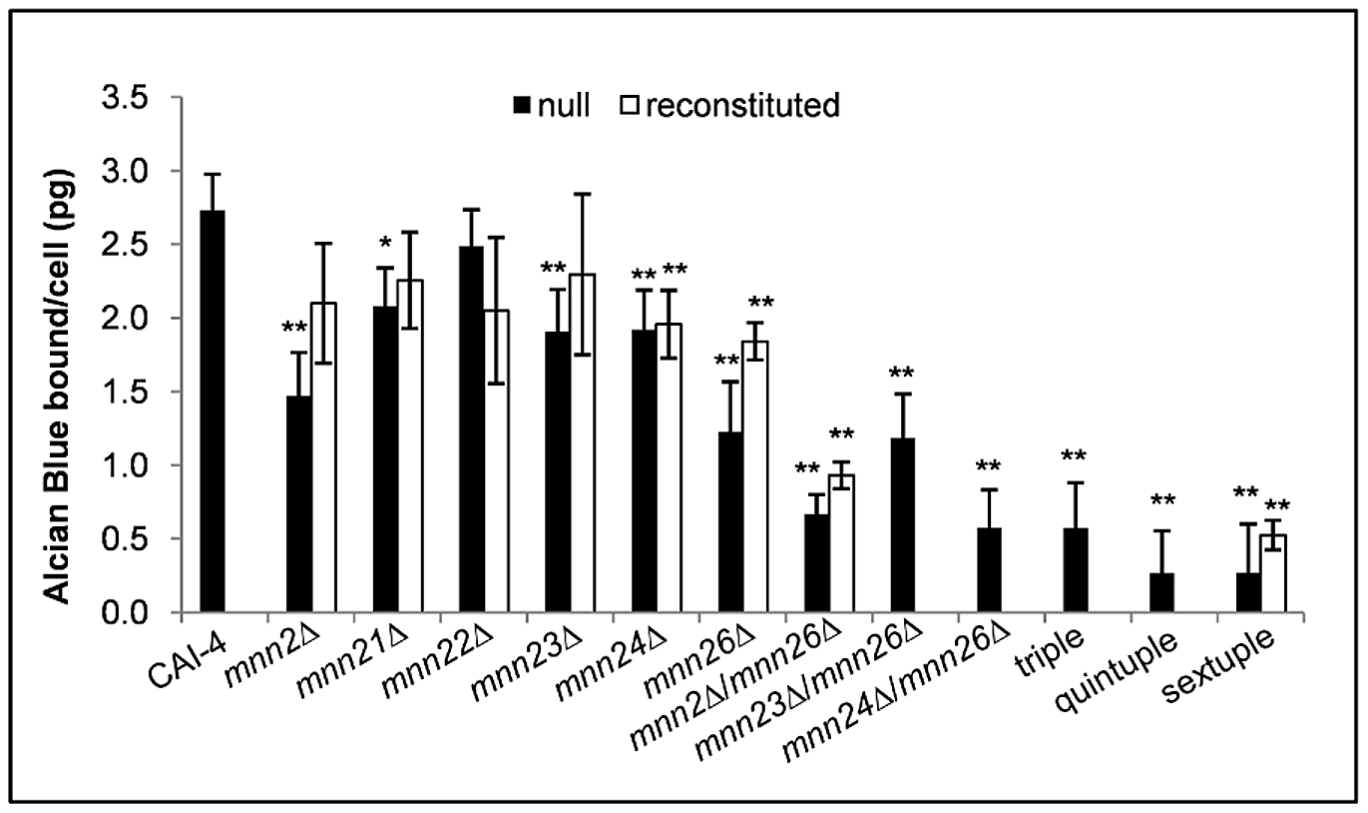
The *MNN2* gene family provides the mannan scaffold to which the phosphomannan is attached. Mutants were incubated in 30 µg/ml Alcian Blue for 10 min and the amount of dye bound to the cell wall estimated by absorbance. Data represent the mean amount of dye bound per cell ± SD from 6 independent experiments, *p<0.05, **p<0.01.

The phosphomannan moiety of the *N*-linked mannan is attached to the branched mannan via α1,2-mannose residues, which extend out from the α1,6-mannose backbone. Therefore, deletion of *MNN2* family members was predicted to affect the phosphomannan content of the cell wall. Alcian Blue is a cationic dye, which binds the negatively charged phosphate group of the phosphomannan and its binding to cells can be used to give an estimate of the cell wall phosphomannan content. Deletion of *MNN2* or *MNN26* significantly reduced the ability of cells to bind Alcian Blue (Figure 1), suggesting that they are a prerequisite for phosphomannan incorporation, while deletion of other genes showed less of a reduction in phosphomannan content (Figure 1). The binding of Alcian Blue was further reduced in the *mnn2*Δ/*mnn26*Δ double mutant, suggesting that Mnn2 and Mnn26 do not have redundant functions (Figure 1). Complementation of the mutants with a single copy of the respective gene partially restored Alcian Blue binding (Figure 1), in agreement with previous studies where restoration of the *mnn4*Δ mutant again only partially restored Alcian Blue binding [34]. Deletion of all six *MNN2* orthologues totally abrogated Alcian Blue binding, suggesting that the cell wall was devoid of phosphomannan and that all measurable phosphomannan is part of the *N*-mannan outer chain structure (Figure 1).

### Deletion of *MNN2* genes results in altered cell wall composition, reduced cell wall integrity and temperature sensitivity

**Figure 2:**
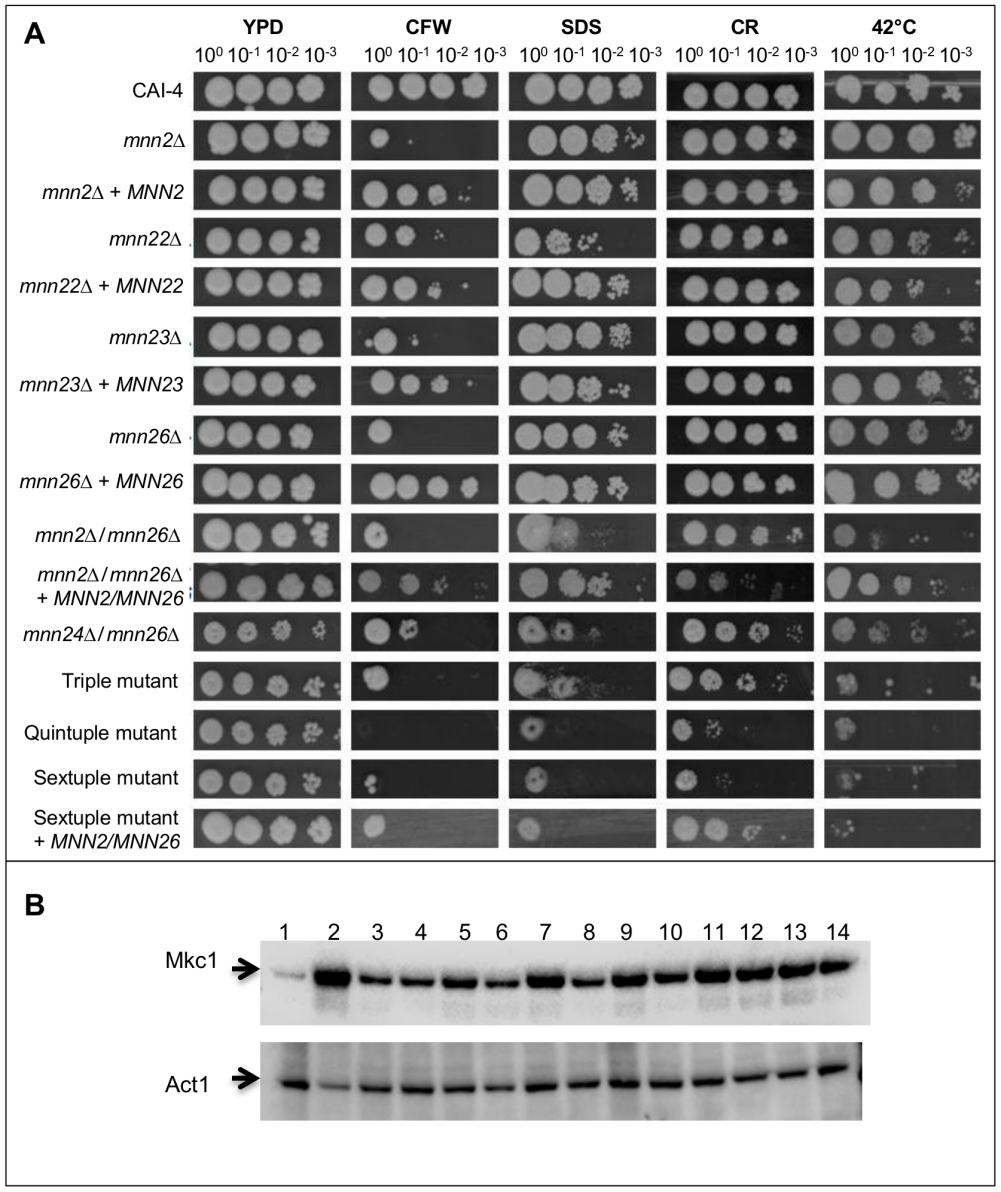
The *MNN2* gene family is required for cell wall integrity. (**A**) Stationary phase cultures were diluted to OD_600_ 0.5 in YPD and 3 µl from 1∶10 serial dilutions spotted onto YPD agar plates containing either 100 µg/ml CFW, 0.03% SDS, 100 µg/ml Congo Red (CR) and incubated at 30°C for 48 h, or spotted on YPD agar and incubated at 42°C. (**B**) Activation of the cell wall salvage pathway was determined by western blot on crude protein extracts prepared from exponentially growing cells. The PhosphoPlus P44/42 antibody detects phosphorylated (activated) Mkc1. As a positive control for Mkc1 activation wild type (CAI-4+CIp10) cells were exposed to 0.0032 µg/ml caspofungin for 10 min. Lane 1 wild type +CIp10, lane 2 wild type+caspofungin, lane 3 *mnn2*Δ, lane 4 *mnn2*Δ+*MNN2*, lane 5 *mnn26*Δ, lane 6 *mnn26*Δ+*MNN26*, lane 7 *mnn2*Δ/*mnn26*Δ, lane 8 *mnn2*Δ/*mnn26*Δ+*MNN2/MNN26*, lane 9 *mnn23*Δ*/mnn26*Δ, lane 10 *mnn24*Δ/*mnn26*Δ, lane 11 triple mutant, lane 12 quintuple mutant, lane 13 sextuple mutant, lane 14 sextuple mutant+*MNN2/MNN26.*

To determine how deletion of the *MNN2* gene family members affected the composition of the cell wall, the quantities of the three main cell wall polysaccharides (chitin, glucan and mannan) were quantified by HPLC sugar composition analysis (Table 1). Deletion of single genes resulted in a 20–50% decrease in the relative proportion of mannan in the cell wall, with *mnn2*Δ and *mnn26*Δ showing the greatest reduction (51.7 and 49.3% reduction, respectively). However, deletion of multiple genes resulted in a 70% reduction in the relative proportion of mannan in the cell wall. The relative amount of glucan in the cell wall remained consistent in all strains. The reduction in the proportion of mannan content in the cell walls of the triple, quintuple and sextuple mutants was accompanied by a 4-fold increase in chitin. The changes in the proportions of cell wall components corresponded with an increased sensitivity to Calcofluor White (CFW), Congo Red and SDS, suggesting that cell wall integrity in these mutants was compromised (Figure 2A). Assessment of Mkc1 activation (phosphorylation) by Western analysis confirmed that the strains which displayed increased sensitivity to agents, had a constitutively activated cell wall salvage pathway (Figure 2B). Both the quintuple and sextuple mutants were also severely affected in their ability to grow at 42°C (Figure 2A).

**Table 1:** Relative proportions of carbohydrates in the cell wall extracted from the *MNN2* gene family mutants.

Strain	Chitin	Glucan	Mannan
CAI-4 CIp10	3.0 (±1.2)	73.2 (±0.7)	23.8 (±1.9)
*mnn2*Δ	4.6 (±0.02)	84.0* (±1.1)	11.5** (±1.1)
*mnn21*Δ	4.0 (±0.7)	79.8 (±3.2)	16.2** (±2.5)
*mnn22*Δ	4.9 (±4.4)	78.3 (±8.8)	16.9** (±4.5)
*mnn23*Δ	1.9 (±1.5)	85.4** (±3.8)	12.7** (±2.3)
*mnn24*Δ	4.8 (±1.6)	80.8 (±3.3)	14.4** (±1.7)
*mnn26*Δ	3.5 (±1.3)	84.4* (±0.6)	12.1** (±0.7)
*mnn2*Δ/*mnn26*Δ	7.9 (±2.6)	82.6 (±1.9)	9.6** (±2.1)
*mnn23*Δ/*mnn26*Δ	4.8 (±3.3)	85.1 (±0.9)	10.1** (±2.5)
*mnn24*Δ/*mnn26*Δ	3.9 (±0.4)	88.8** (±3.1)	7.4** (±2.8)
triple mutant	11.7* (±4.3)	79.0 (±5.6)	9.3** (±2.2)
quintuple mutant	10.1 (±1.7)	83.8 (±0.9)	6.1** (±2.6)
sextuple mutant	12.2* (±6.0)	80.8 (±4.3)	7.1** (±1.9)
*mnn2*Δ+*MNN2*	3.3 (±0.9)	79.3 (±1.4)	17.3 (±2.3)
*mnn21*Δ+*MNN21*	3.7 (±0.1)	82.2 (±0.8)	14.1 (±0.7)
*mnn22*Δ+*MNN22*	4.4 (±2.0)	82.5 (±1.0)	13.1* (±1.0)
*mnn23*Δ+*MNN23*	6.6 (±6.1)	76.7 (±11.0)	16.7 (±4.9)
*mnn24*Δ+*MNN24*	2.4 (±0.03)	83.4 (±0.4)	14.2 (±0.4)
*mnn26*Δ+*MNN26*	3.5 (±1.0)	84.1 (±5.1)	12.4* (±4.2)
*mnn2*Δ*/mnn26*Δ+*MNN2/MNN26*	3.9 (±1.5)	81.5 (±4.3)	14.7 (±2.9)
sextuple mutant+*MNN2/MNN26*	13.4 (±3.2)	77.6 (±4.8)	9.0** (±1.7)

* p<0.05,

** p<0.01.

### The *MNN2* gene family regulates mannan fibril length

**Figure 3:**
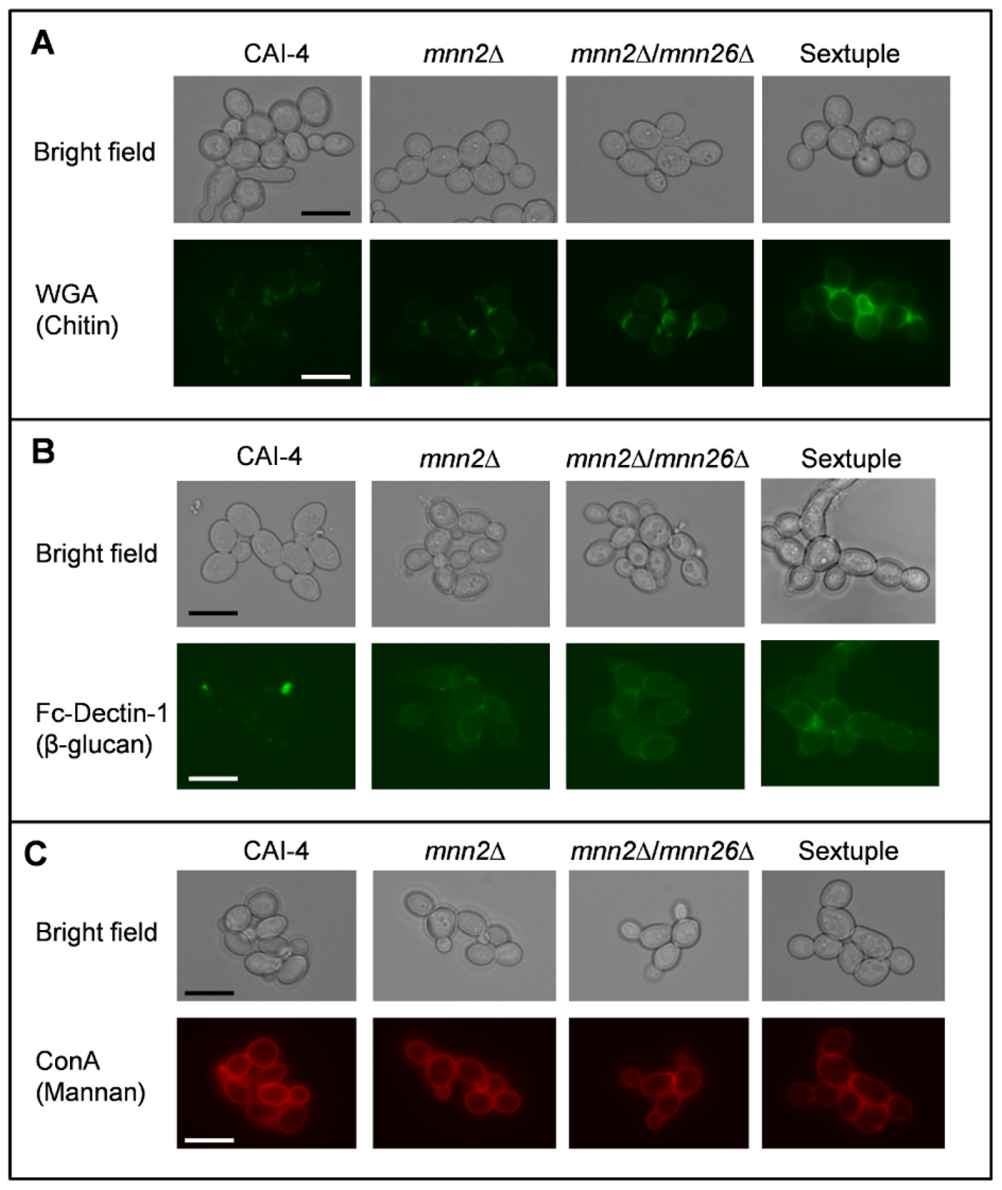
Deletion of *MNN2* gene family members increases chitin content and β-glucan exposure. Exponentially growing cells were stained with **A**) WGA-FITC to visualise chitin **B**) Fc Dectin-1 to visualise β-glucan and **C**) ConA-TR to visualise mannan. Scale bar represents 10 µm.

**Figure 4:**
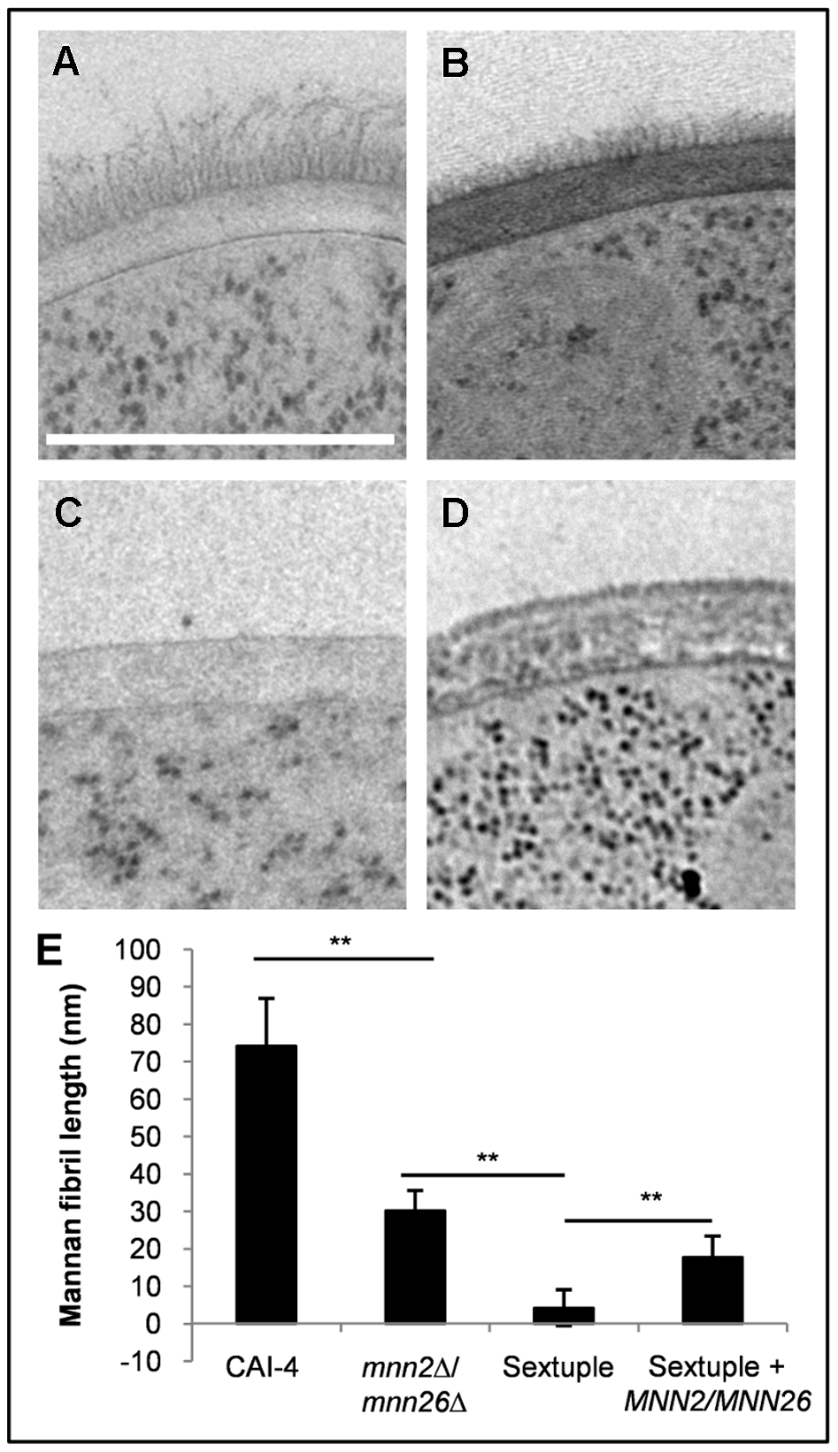
The Mnn2 family of mannosyltransferases regulates mannan fibril length. Electron micrographs showing the ultrastructure of the cell walls of the (**A**) wild type (CAI-4+CIp10), (**B**) *mnn2*Δ/*mnn26*Δ, (**C**) sextuple mutant, (**D**) sextuple mutant+*MNN2/MNN26* strains. The scale bar represents 500 nm. (**E**) Mannan fibril length was measured in 8 randomly selected cells. Each cell was measured 10 times in different locations. Data represent the means ± SD, **p<0.01.

To visualise changes in the cell wall structure, we performed two types of microscopic analyses. First, fluorescence microscopy was performed on fixed cells stained with either wheat germ agglutinin (WGA)-FITC, concanavalin A (ConA)-TR or Fc-Dectin-1 to assess whether there were any significant changes in the exposure of chitin, mannan or β-glucan on the *C. albicans* cell surface (Figure 3). Fluorescence microscopy showed that WGA-FITC stained the lateral cell wall of the sextuple mutant, but only stained the chitin exposed in the bud scars of the parental control strain (Figure 3A). This reflects the large size of WGA that prevents deep penetration of the wall so that only chitin that is exposed near the surface of the cell wall is accessible to this lectin. The increased amount of WGA-FITC staining of the cell wall in the sextuple mutant suggested that the chitin was no longer restricted to the inner cell wall layer, but became distributed throughout the depth of the cell wall. Previous studies have also shown that cells with increased chitin but normal mannan levels bind WGA-FITC more readily [20], confirming that the observed increase in WGA binding was not the result of increased WGA penetration due to the loss of the outer mannan layer. β-glucan exposure was visualised by FITC labelled Fc-Dectin-1 binding. Binding to wild type cells was extremely low, suggesting that the outer mannan layer shielded the β-glucan (Figure 3B). However, the *mnn2*Δ/*mnn26*Δ and the sextuple mutants exhibited an increased patchy binding of Fc-Dectin-1 around the cell periphery (Figure 3B), suggesting that there was increased exposure of β-glucan. This increased exposure was hypothesised to result from the loss of mannan from the outer cell wall. However, ConA-TR staining of the mutants suggested that the cell wall periphery still contained a significant amount of residual mannan (Figure 3C) that was likely to be derived from the remaining *N*-mannan core and from *O*-mannans. Therefore, transmission electron microscopy (TEM) of high-pressure frozen, freeze-substituted samples was used to visualise the ultrastructure of the cell wall (Figure 4). No significant difference in the thickness of the inner cell wall layer between the parental control strain and the sextuple mannosylation mutants were observed in electron micrographs (Figure 4 A–D). However, the inner cell wall layer of the parental control strain was surrounded by an external layer of mannosylated proteins, which appeared as 74±13 nm fibrils extending out from the chitin-β-glucan layer in the electron micrographs (Figure 4A, E). These fibrils were significantly shorter (30±5 nm) in the *mnn2*Δ/*mnn26*Δ mutant (Figure 4B, E). The sextuple mutant, however, was almost completely devoid of long mannan fibrils, with only occasional, short (4±4 nm) fibrils observed on the cell wall surface (Figure 4C, E). Reintegration of both *MNN2* and *MNN26*, was sufficient to establish small (18±7 nm) fibrils, which covered the complete cell wall surface (Figure 4D, E).

### Physical and chemical analysis of the *mnn2* mutant cell walls by GPC analysis and NMR

**Figure 5:**
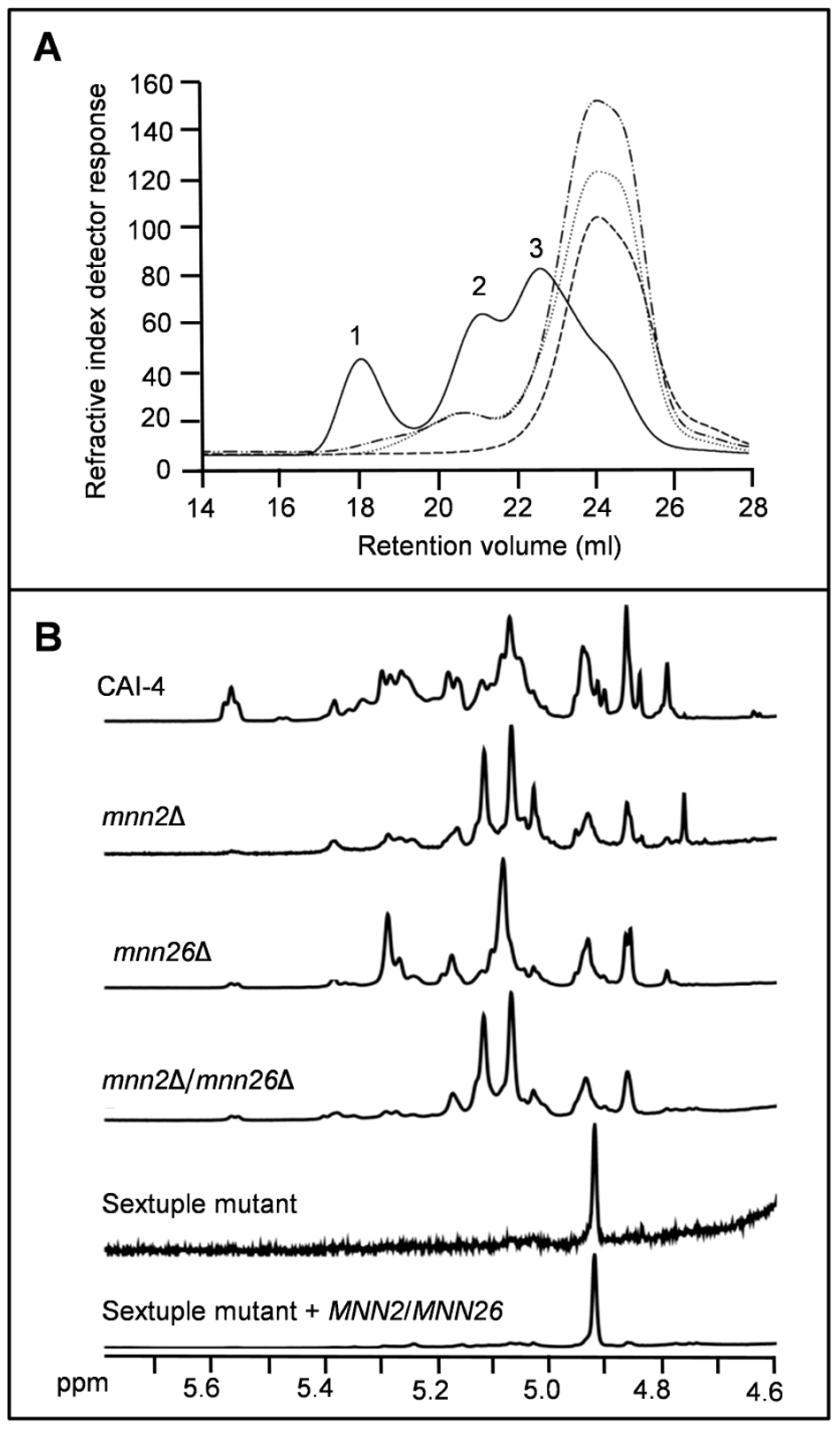
Deletion of *MNN2* gene family members results in a lower molecular weight and less complex *N*-mannan. (**A**) *C. albicans N*-mannans were extracted from cells and analysed by GPC. Solid line represents wild type (CAI-4+CIp10), dotted line represents mnn26Δ, dashed line represents mnn2Δ/mnn26 and the dashed and dotted line represents mnn24Δ/mnn26Δ. The numbers designate the peaks discussed in the text. (**B**) Representative proton NMR spectra for the *N*-mannan extracted from wild type, *mnn2*Δ, *mnn26*Δ, *mnn2*Δ/*mnn26*Δ, sextuple mutant and the reconstituted sextuple mutant.

To further investigate how deletion of genes encoding members of the Mnn2 mannosyltransferase family affected the structure of the mannan fibrils, *N*-mannans extracted from seven of the mutants (*mnn2*Δ, *mnn21*Δ, *mnn26*Δ, *mnn2*Δ/*mnn26*Δ, *mnn24*Δ/*mnn26*Δ, the triple, and the sextuple mutants) and the respective reconstituted control strains were analysed by gel permeation chromatography (GPC) and proton nuclear magnetic resonance (NMR). We failed to isolate sufficient purified *N*-mannan for analysis from all of the multiple mutants by gel permeation chromatography (GPC) due to the marked reduction in cell wall mannan content. However, we obtained sufficient mannan from all the mutants for NMR and enough mannan from *mnn26*Δ, *mnn2*Δ/*mnn26*Δ and *mnn24*Δ/*mnn26*Δ to perform GPC. GPC analysis for the parental control mannan confirmed that the mannan/mannoprotein had a trimodal polymer distribution (Figure 5A). The first peak had an average molecular weight (Mw) of 40×10^5^ Da, but only represented 17% of the total cell wall mannan/mannoprotein. The third peak had an average Mw of 1.7×10^5^ Da and represented 60% of the mannan/mannoprotein. In comparison, the mannan from the *mnn26*Δ and *mnn24*Δ/*mnn26*Δ mutants showed a bimodal polymer distribution, and the mannan/mannoprotein from *mnn2*Δ/*mnn26*Δ eluted primarily as a single peak resulting from a loss of the high molecular weight mannan (peaks 1 and 2, Figure 5A). In addition, the third peak of all the *mnn2* mutants was shifted to the right, suggesting that the mannan/mannoprotein in the mutants had a lower Mw than the parental control strain (Figure 5A). Single peak analysis confirmed that the average Mw of the mannan/mannoprotein of the *mnn26*Δ, *mnn24*Δ/*mnn26*Δ and *mnn2*Δ/*mnn26*Δ mutants were 71.6, 62.3 and 93.2% of the parental control strain (CAI-4+CIp10 10.3×10^5^ Da, *mnn26*Δ 2.9×10^5^ Da, *mnn24*Δ/*mnn26*Δ 3.9×10^5^ Da and *mnn2*Δ/*mnn26*Δ 0.7×10^5^ Da). Therefore, the mannans produced by the mutants are less complex and more uniform than those from the wild type parental control strain. Treatment of the mannan/mannoprotein with pronase to digest the protein attached to the mannan confirmed that all mutants had less mannan associated protein, with *mnn2*Δ/*mnn26*Δ showing the most significant decrease (Table S1).

**Figure 6:**
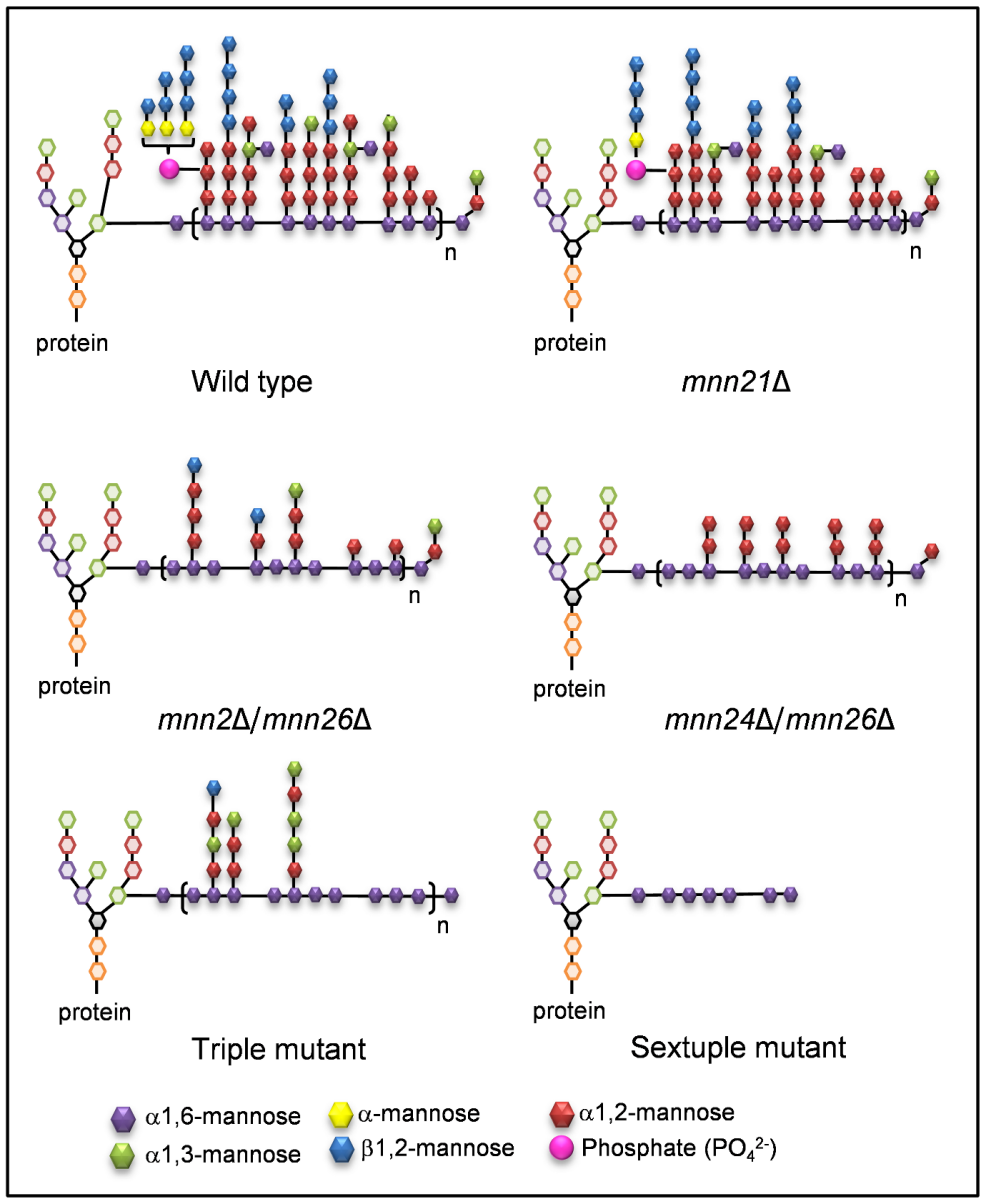
Predicted structures of the *C. albicans N*-mannan in the respective mutants as identified by NMR. The *N*-mannan structure is based on the work by Shibata and colleagues [26], [43], [45], [48].

When analysed by proton NMR, the mannans from the mutants were classified as belonging to four structural groups (Figure 5B and Table S2). The mannans in Group 1 were most structurally similar to the wild type parental control strain. This group was comprised of the reconstituted control strains of the single and double mutants, suggesting that the expression of a single copy of the respective gene partially or fully complemented the structural defects observed in the mutants (data not shown). The *mnn21*Δ mutant also formed part of Group 1, but showed a significant reduction in phosphomannan content compared to the parental control strain. This reduction was mainly due to the loss of mono- and di-phosphate groups (Mα1-PO_4_ and Mβ1-2Mα1-PO_4_) and exhibited reduced resonances at 5.55, 5.47, 4.91 and 4.83 ppm, but the mutant retained some long phosphate chains ((Mβ1)_n_-2Mα1-PO_4_). In addition, the *N*-mannan of the *mnn21*Δ mutant also contained less α1,3-mannose residues (expressed as a reduction in the area of the resonance at 5.33 ppm), suggesting that the α1,2-mannose preceding the α1,3-mannose was missing (Figure 6 and data not shown). Group 2 consisted of the *mnn2*Δ and *mnn2*Δ/*mnn26*Δ mutants. The *N*-mannan from these mutants showed a significant reduction in α1,2-mannose side chains (represented by reduction in resonances between 5.29 and 5.15 ppm) and reduced phosphomannan. The larger resonance at 5.117 ppm suggested that there were regions of un-substituted α1,6-mannose residues in the backbone. There was still a strong signal at 5.068 ppm, which suggested that there were substituted α1,6-mannose units interspersed in the un-substituted regions. The area of these two resonances was roughly equivalent, indicating that the quantities of substituted and un-substituted α1,6-mannose in this mutant were similar (Figure 5B and Figure 6). The mutants in Group 3 (*mnn26*Δ, *mnn24*Δ/*mnn26*Δ) displayed reduced levels of α1,3-mannose in the outer chain, with the *mnn24*Δ/*mnn26*Δ mutant being almost completely devoid of α1,3-mannose. The strongest NMR resonance in these mutants was shifted from 5.067 ppm to 5.088 ppm, suggesting significant loss in α1,2-mannose side chains that are linked to α1,6-mannose residues, which, in turn, were linked to other substituted α1,6-mannose residues. A significant peak at 5.28 ppm was still present, so it is possible that these side chains contained two α1,2-mannose units (Figure 5B, Figure 6 and data not shown). Group 4 was represented by the triple and sextuple mutants and the sextuple reintegrated control strain. The triple mutant (*mnn2*Δ/*mnn22*Δ/*mnn23*Δ) showed reduced phosphomannan, and β1,2-mannose residues. In addition, resonances at 5.067, 5.088, 5.107 and 5.120 ppm exhibited reduced intensity, which correlated with the appearance of a resonance at 4.92 ppm (data not shown). Taken together, these changes in the NMR spectrum suggest that there were significant regions of un-substituted α1,6-backbone between α1,6-backbone branch points. However, in contrast to the mutants in Groups 2 and 3, the triple mutant displayed an increased level of α1,3-mannose, suggesting that in the absence of α1,2-mannose, α1,3-mannose may be incorporated into the outer chains (Figure 6). Deletion of all six putative *MNN2* gene family members resulted in an *N*-mannan structure comprised predominantly of α1,6-mannose, suggesting that only the backbone remained (Figure 5B, Figure 6). Reintegration of *MNN2* and *MNN26* in the sextuple mutant was not sufficient to restore outer chains on the backbone, but did result in a moderate increase in side chain initiation, which appeared to correlate with increased levels of α1,6-mannan (Figure 5B). The backbone was completely un-substituted in the sextuple mutant making it likely that the increased incorporation of α1,3-mannan into the outer chains only occurs when there is residual α1,2-mannosyltransferase activity. Therefore, only α1,2-mannan is attached to the α1,6-backbone.

### Loss of α1,2-mannan inhibits immune recognition of *C. albicans* by PBMCs

**Figure 7:**
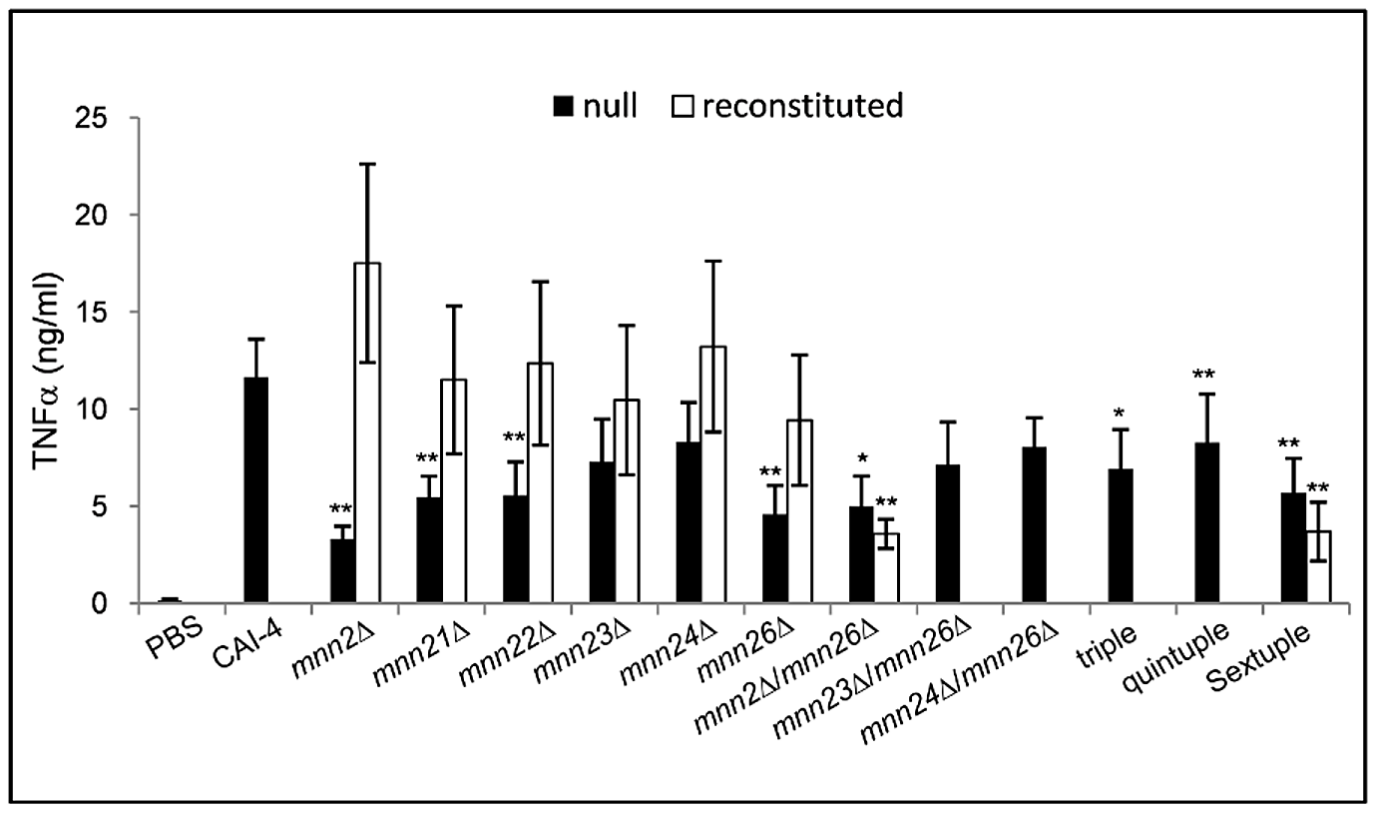
Deletion of *MNN2* gene family members reduces immune recognition. PBMCs were co-incubated with heat-killed *C. albicans* cells at an MOI of 0.4 for 24 h. The concentration of secreted cytokines was measured by ELISA. Data represent the means ± SEM from 8 independent experiments, *p<0.05, **p<0.01.

Having determined that the members of the *MNN2* gene family are required for the addition of α1,2-mannan into the *N*-mannan outer chains, we assessed whether the loss of α1,2-mannan affected immune recognition by peripheral blood monocytes (PBMCs). Mannan is an important PAMP recognised by multiple PRRs. As a result, disruption of mannan biosynthesis is likely to have a significant impact on fungal immune recognition. To determine how the loss of α1,2-mannan affected immune recognition, heat-killed *C. albicans* cells were co-incubated with PBMCs for 24 h and the cytokine profile determined as a measure of immune cell activation. Deletion of *MNN2* resulted in a 67% reduction in TNFα secretion from PBMCs, while deletion of *MNN21*, *MNN22* or *MNN26* reduced TNFα production by 50% (Figure 7). Deletion of multiple *MNN2* gene family members also reduced TNFα production to varying degrees (Figure 7). PBMCs also secreted significantly less IL-1β when co-incubated with the various *MNN2* gene family mutants compared to the parental control strain (Figure S2A). On the other hand, secretion of IL-6 was only significantly reduced when PBMCs were co-incubated with *mnn23*Δ/*mnn26*Δ, the quintuple and the sextuple mutants (Figure S2B). The PBMCs elicited a greater response to the quintuple and sextuple mutants compared to the double and single mutants, suggesting that complete removal of the branched *N*-mannan, in conjunction with heat killing the cells, exposed other immune stimulatory epitopes. We therefore determined the effect of different commonly used methods of killing cells for immunological assays on the integrity of the cell wall of the parental control strain by TEM (Figure S3). Heat killed cells showed the greatest cell wall disruption, although the general architecture of the inner and outer cell wall layers was still obvious. The cell walls of thimerosal and UV-killed cells exhibited less visible perturbation of the wall structure (Figure S3). We stimulated PBMCs with a selection of mutants that had been killed by thimerosal. In agreement with the TEM images, the thimerosal killed cells elicited a lower immune response than heat killed cells (Figure S4), suggesting that a large portion of the detected immune response of heat killed cells was due to disruption of the cell wall and exposure of the underlying β-glucan. However, TNFα secretion of the mannosylation mutants was similar to that of heat-killed cells with the mannosylation mutants stimulating significantly less of a proinflammatory response than the parental control strain (Figure S4).

### The *MNN2* gene family is required for virulence

**Figure 8:**
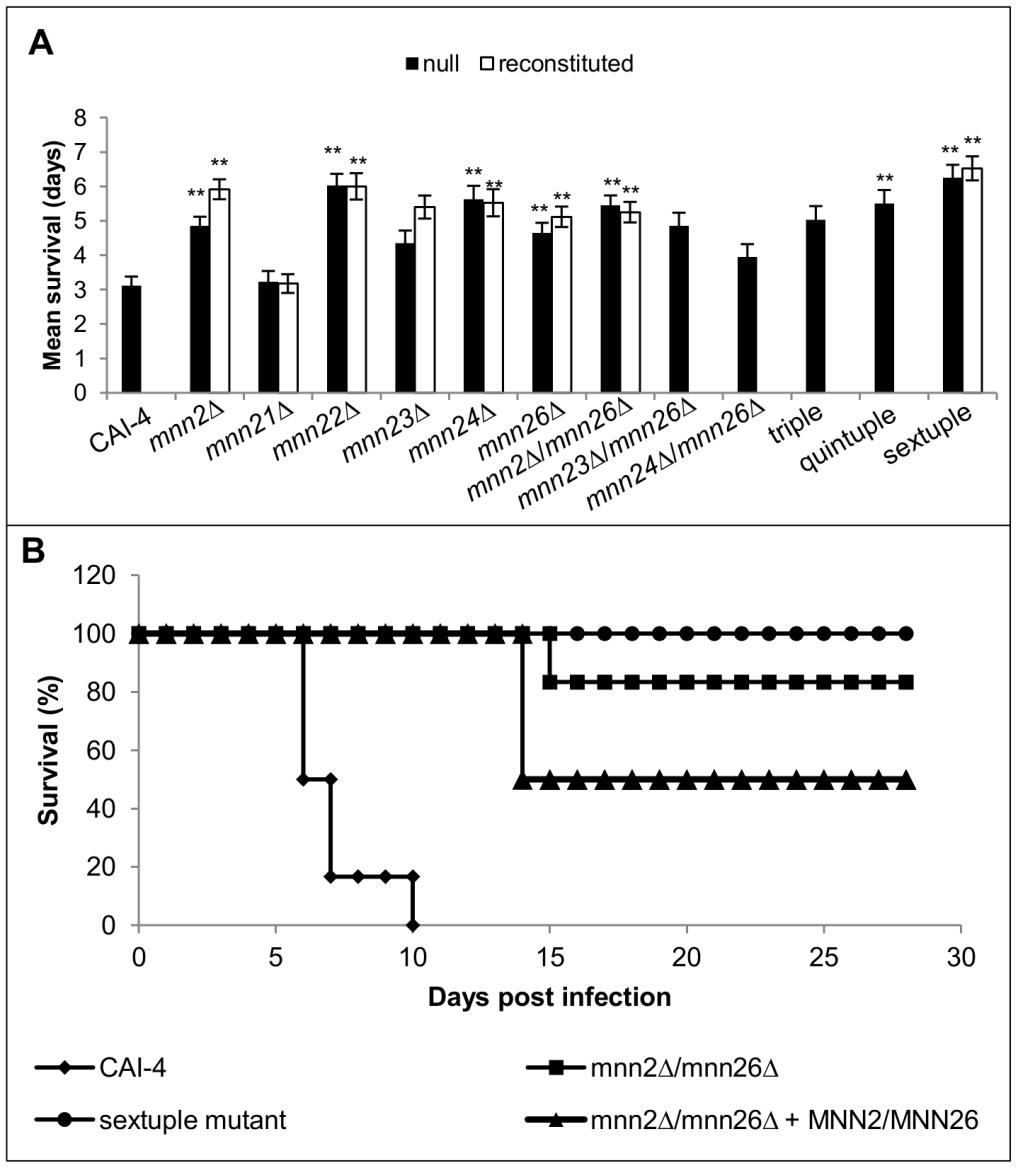
Mnn2 members are required for virulence of *C. albicans*. (**A**) Mutants were screened for virulence defects in the *G. mellonella* infection model. Larvae were infected with 2.5×10^5^ *C. albicans* cells and incubated at 37°C. Data represent the mean larvae survival times ± SD from 3 independent experiments, *p<0.05, **p<0.01. (**B**) wild type (CAI-4+CIp10), *mnn2*Δ/*mnn26*Δ, *mnn2*Δ/*mnn26*Δ+*MNN2/MNN26* and the sextuple mutant were tested for their ability to cause infection in the 28 day mouse model of systemic infection.

To determine how the loss of α1,2-mannan seen in the mutants of the *MNN2* gene family affected the virulence of *C. albicans*, we first assessed the virulence of all the mutants using the *Galleria mellonella* infection model. Inoculation of larvae with 2.5×10^5^ cells resulted in a mean survival time of 3 days for the parental control strain. However, larvae infected with *mnn2*Δ, *mnn22*Δ, *mnn24*Δ, *mnn2*Δ/*mnn26*Δ, the quintuple and the sextuple mutant had mean survival times between 6–7 days, suggesting that these strains were less virulent than the parental control strain (Figure 8A).

Immunity in *G. mellonella* is a useful adjunct model for screening a large number of strains for differences in virulence, however immunity in this organism is predominantly mediated by haemocytes, and does not reflect the full immunological response of a mammalian host. Selected mutants were therefore also examined in a systemic mouse model of infection. The *mnn2*Δ/*mnn26*Δ mutant, its reconstituted control strain and the sextuple mutant were tested in the 3-day and 28-day murine infection model to establish the involvement of α1,2-mannan in both early infection processes and for maintenance of infection. In the 3-day infection model, virulence is expressed as an “outcome score”, which is related to weight loss and kidney fungal burdens [35]. Accordingly, the higher the outcome score the greater the kidney burden and weight loss, and therefore the greater the virulence of the strain [35]. The *mnn2*Δ/*mnn26*Δ and sextuple mutants had significantly reduced outcome scores (p = 0.004 and p = 0.002, Table 2), which correlated with reduced kidney burdens and reduced weight loss, underlining that these mutants were attenuated in virulence compared to the parental control strain. In the 28-day infection model, 80% of mice survived infection with the *mnn2*Δ/*mnn26*Δ mutant, with only 20% succumbing to infection on day 14 (Figure 8B). In contrast, all mice infected with the sextuple mutant survived the infection (Figure 8B). The kidney burdens for *mnn2*Δ/*mnn26*Δ and the sextuple mutant at 28 days post infection were 3.9±3.0 and 4.9±1.9 log_10_ CFU/g, respectively, suggesting that the mutants were not completely eradicated from the mice. The *mnn2*Δ/*mnn26*Δ reconstituted control strain had a similar outcome score and kidney burden to *mnn2*Δ/*mnn26*Δ, but showed increased weight loss and only 50% survival in the 28-day infection model, indicating that ectopic expression of a single copy of *MNN2* and *MNN26* was insufficient to restore virulence to wild type levels (Table 2, Figure 8B).

**Table 2:** Deletion of *MNN2* gene family members attenuates virulence in the 3-day murine infection model.

	Kidney burden (log_10_CFU/g)		% weight change		Outcome score	
Strain	Mean	Stdev	Mean	Stdev	Mean	Stdev
CAI-4+CIp10	5.0	0.3	−8.6	3.9	9.3	2.1
mnn2Δ/mnn26Δ	4.2	0.7	−1.6	2.7	5.0	1.1
mnn2Δ/mnn26Δ+*MNN2/MNN26*	3.3	0.7	−0.8	1.4	3.7	1.0
Sextuple mutant	4.3	0.4	−2.5	2.0	5.5	1.2

## Discussion

The fungal cell wall is the first point of contact between the invading pathogen and the host immune system. All the major cell wall carbohydrates have been shown to act as PAMPs that activate and modulate the host's immune response to the invading pathogen mediated via interactions with specific PRRs [5], [13], [17]–[18], [36]. Previous studies using *C. albicans* mannosylation mutants have confirmed that both *O*- and *N*-mannan structures are important for the recognition of *C. albicans* by monocytes, macrophages, neutrophils and dendritic cells and established that *N*-linked mannan was an agonist of the MMP and DC-SIGN [7]–[10], [37]–[39]. However, most PRRs bind short oligosaccharides and the precise carbohydrate epitopes that these PRRs recognise are unknown. Here, we expand on these studies by characterising the role(s) of the α1,2-mannose residues from the *N*-mannan in immune recognition and fungal virulence through deletion of the *C. albicans MNN2* gene family.

Deletion of *MNN2* gene family members resulted in increased cell aggregation - a common phenotype of glycosylation mutants [7], [9]–[11]. Cell aggregation can result from enhanced binding of adhesions within the cell wall, or from inactivation of hydrolytic enzymes required to digest the cell wall at the bud site. High concentrations of sugars and the addition of Congo Red have been shown to compete with adhesin binding and disrupt Als5 amyloid formation decreasing cell aggregation [40]–[41]. However, addition of Congo Red, or high concentrations (20%) of glucose, did not disrupt the cell aggregates (data not shown), suggesting that this was not the result of Als5 amyloid formation or increased exposure of Als1 binding sites. Therefore, it is possible that these clumps of cells result from insufficient cell separation due to reduced hydrolytic activity of the enzymes responsible for cell wall cleavage after cell division.

The sextuple mutant has severely truncated mannoprotein fibrils, however the presence of residual mannan was confirmed by positive ConA-TR staining and HPLC analysis which showed that mannan still comprised approximately 7% of the relative cell wall dry weight. This low mannan proportion may be attributed to the *O*-mannan and remaining α1,6-backbone from the *N*-mannan. The use of high-pressure freezing and freeze substitution to prepare samples for TEM has enabled better preservation of the ultrastructure of the mannan fibril layer, which is not well preserved by traditional chemical fixation protocols. This allowed us to demonstrate that deletion of specific *MNN2* gene family members generated mannan fibrils of varying length. Therefore, it is possible that each fibril may represent one *N*-mannan covalently attached to a cell wall protein with the length of the fibril representing the outer chain incorporating the α1,6-backbone and its side chains extending away from the cell. Assuming that one mannose unit is approximately 0.5 nm and the bond length between each mannose residue is approximately 0.15 nm, and recognising that alpha-linked complex mannan polymers are very likely to be linear molecules, then a mannan fibril of 100 nm can be estimated to have a backbone comprised of approximately 150 α1,6-mannose units. The fibrils in the *mnn2*Δ/*mnn26*Δ mutant were approximately 30 nm in length, which would correspond to approximately 46 α1,6-mannose units. The *N*-mannan from the reconstituted sextuple mutant contained more α1,6-mannose than the sextuple mutant and fibrils were only observed on the surface of the reconstituted strain. The NMR spectra suggest that the reconstituted strain had more side chain residues than the mutant. Therefore, it is possible that these small side chain residues are sufficient to stabilise the α1,6-backbone, allowing increased extension of the backbone by Mnn9.

**Figure 9:**
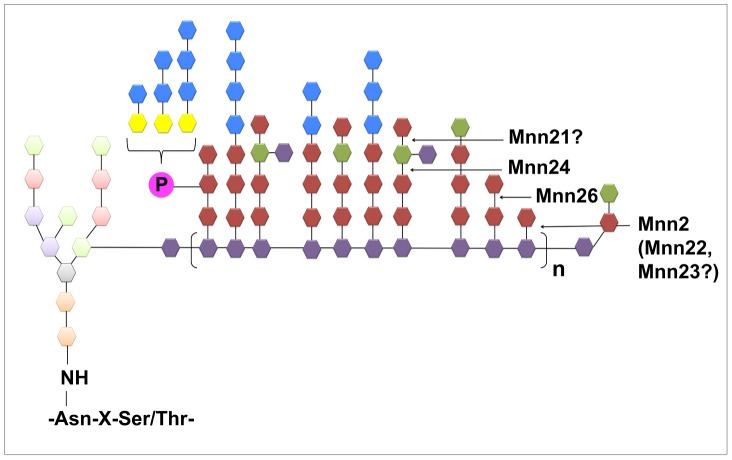
Hypothetical model for the actions of the six Mnn2 mannosyltransferases. See text for details.

Previous studies have proposed a model for *C. albicans N*-mannan structure [26], [42]–[47] and additional experiments combining mild acetolysis, HPLC and 2D NMR have identified the composition and linkage of the *N*-mannan side chains [48]. More recently, Lowman and colleagues described a method combining 1D and 2D COSY and NOSEY NMR with published chemical shift data for specific side chains to assign resonances to non-degraded intact *N*-mannans from yeast [49]. This approach permitted rapid identification of structural motifs for acid-liable and acid-stable fractions of *N*-mannan. Using this method we compared changes in the side chain motifs in our mutants and suggest structures for the different *N*-mannans expressed in each of the mutants (Figure 6). From our data, we hypothesise that Mnn2 is the major α1,2-mannosyltransferase responsible for the addition of the initial α1,2-mannose residue onto the α1,6-backbone, as deletion of *MNN2* significantly reduced α1,2-mannose content. However, deletion of *MNN2* alone did not prevent incorporation of α1,2-mannose into the side chains, suggesting a degree of functional redundancy within the family. The triple mutant displayed significant regions of un-substituted backbone compared to the *mnn2*Δ mutant. Therefore, it is likely that Mnn22 and Mnn23 initiate outer chain branching in the absence of Mnn2. Mnn23 has already been described as an Mnn5 orthologue because it can functionally complement the *S. cerevisiae mnn5*Δ mutant [50]. However, the mannans in *S. cerevisiae* and *C. albicans* differ significantly, so it is possible that Mnn23 has both Mnn2 and Mnn5 activity in *C. albicans*. Deletion of *MNN24* and *MNN26* resulted in a significant decrease of α1,3-mannan in the side chains, which was most obvious in the *mnn24*Δ/*mnn26*Δ double mutant. Therefore, we suggest a model (Figure 9) where Mnn26 is required to add the second α1,2-mannose residue onto the side chain, with the third α1,2-mannose attached by Mnn24 to generate an [α1,2-mannose]_3_ oligosaccharide to which α1,3-mannose residues are finally attached by members of the Mnn1 family of glycosyltransferases. In agreement with this, deletion of *MNN2* and *MNN26* resulted in an almost complete loss of acid-liable side chains (phosphomannan), which are predominantly attached to *N*-mannan via the second or third α1,2-mannose in the side chain [51], confirming that the *mnn2*Δ and *mnn26*Δ mutants do not normally possess side chains longer than two α1,2-mannose residues. Deletion of *MNN21* did not significantly alter the NMR spectra, suggesting little change in side chain composition. It is possible that Mnn21 contributes to *N*-mannan biosynthesis through capping some of the α1,3-mannose units with α1,2-mannose residues (Figure 9).

Single mutants of the *MNN2* gene family elicited a lower immune response when co-incubated with PBMCs, suggesting that these mutants lacked important PAMPs required for immune recognition. On the other hand, heat-killing the sextuple mutant resulted in a increase in the production of pro-inflammatory cytokines compared to thimerosal killed cells, suggesting that more β-glucan was exposed upon heat treatment due to the reduced integrity of the cell wall. Staining exponentially growing cells with soluble Fc-Dectin-1 confirmed that there was more β-glucan exposed on the surface of the sextuple mutant than the parental control strain. However, PBMCs elicited a significantly reduced pro-inflammatory response when the sextuple mutant was killed by thimerosal, which maintains cell wall integrity, suggesting that in these assays conditions mannan and not β-glucan is the major PAMP recognised by the PBMCs. These results are in agreement with previously published data for the *och1*Δ mutant which has significantly increased amounts of β-glucan and reduced amounts of mannan in the cell wall, but still elicits a reduced pro-inflammatory cytokine profile upon co-incubation with PBMCs [7], [16]. The immunological signature of these mutants, therefore, confirm that *N*-mannan is both an immunostimulatory PAMP (in the case of PBMCs) and a shield that masks the highly proinflammatory β1,3-glucan layer (in the case for macrophages) [8], [52]. To determine the importance of the α1,2-mannan in fungal virulence, all mutants were tested for their ability to infect *G. mellonella*. This infection model has been shown to be a valuable tool for screening virulence defects in *C. albicans* strains, with results correlating well with those obtained from murine systemic infection models [53]. Deletion of all *MNN2* gene family members increased the average survival of the moth larvae by 50%, suggesting that α1,2-mannose is important for virulence. The 28-day murine systemic infection model confirmed that the sextuple mutant was attenuated in virulence with 100% of mice surviving infection with the sextuple mutant. Mice infected with *mnn2*Δ/*mnn26*Δ and the sextuple mutant often displayed at least one swollen kidney. Kidney enlargement and unilateral kidney involvement in systemic candidiasis has previously been observed when low inocula of SC5314 are used [54]. After intravenous inoculation of yeast cells into the blood circulation, the majority of *C. albicans* are removed rapidly from the bloodstream with only low levels of *C. albicans* found in the blood 2 h post infection [54]. *C. albicans* cells which reach the kidney are likely to be phagocytosed and cleared by patrolling macrophages, with only the few cells that manage to evade macrophage killing left to establish infection. Increased exposure of β-glucan in the sextuple mutant may enhance macrophage recognition and phagocytosis resulting in rapid clearing and limitation of infection.

Complementation of the mutants with a single copy of the respective gene did not fully restore wild type phenotypes in some of the assays. This is likely to be due to the ectopic expression of the wild type gene at a non-native locus. In addition, our reintegration constructs used 1 kb of DNA upstream from the start codon and 500 bp of terminator sequence. Such constructs may lack some of the regulatory sequences that are required for the normal expression profile of the *MNN2* gene family members. It was noted however that the majority of the cytokine profiles were elevated in the reconstituted strains compared to the mutant. Therefore, expression levels in these strains were sufficient to partially restore mannan biosynthesis to levels where it was sufficient to drive PAMP mediated immune responses but was not sufficient to fully restore virulence.

In conclusion, the addition of α1,2-mannose onto the α1,6-backbone is mediated by the *MNN2* family of α1,2 mannosyltransferases. We present a model (Figure 9) where Mnn2 is required for the addition of the majority of the α1,2-mannose residues onto the backbone of the *N*-mannan which is then elaborated with additional α1,2-mannose units by Mnn26 and Mnn24. Deletion of all six *MNN2* gene family members resulted in the termination of α1,6-mannan elongation, presumably due to reduced stabilisation of the backbone as a result of the loss of mannan side chains, with cells appearing devoid of mannan fibrils. The loss of mannan fibrils from the cell wall surface resulted in increased exposure of the underlying β-glucan layer, and consequential virulence attenuation perhaps due to the mutant being cleared from the kidney by patrolling macrophages as a result of increased Dectin-1 dependent phagocytosis.

This work contributes to the identification of specific carbohydrate epitopes, which are recognised by the PRRs and is relevant in the context of the development of novel immunotherapies and diagnostics. Furthermore, because most fungal mannosyltransferases are not found in mammalian cells, characterisation of the enzymes involved in *N*-mannan biosynthesis may represent novel antifungal drug targets.

## Materials and Methods

### Ethics statement

All animal experimentation conformed to UK Home Office legislation requirements and was approved by the UK Home Office and the Ethical Review Committee of the University of Aberdeen. All animals had free access to food and water throughout the experiments.

### Strains, media and growth conditions

All strains used in this study are listed in Table S3. Strains were maintained as glycerol stocks at −80°C and when required plated onto YPD agar (1% yeast extract, 2% glucose, 2% mycological peptone and 2% agar) and grown at 30°C for 48 h. For the majority of assays, cells were inoculated in YPD, grown to stationary phase at 30°C, 200 rpm and then diluted 1∶100 in fresh YPD and grown to mid exponential phase. For morphology assays, stationary phase YPD cultures were diluted and plated onto Spider medium (1% nutrient broth, 1% mannitol, 0.2% K_2_PO_4,_ 2.5% agar, pH 7.2) and incubated at 30°C, or Dulbecco's modified Eagle medium (DMEM; 1.34% DMEM, 2% glucose, 3.57% HEPES, pH 7) supplemented with 5% foetal calf serum and DMEM in the presence of a 5% CO_2_ atmosphere and incubated at 37°C.

### Construction of mutant strains

All mutants were generated in the CAI-4 background using the mini ura blaster method as previously described [55]. Primers were designed to have 70 bp of homology with the gene of interest and where possible the full open reading frame was deleted from start (ATG) to stop (TAA) (Table S4). To restore the *URA3* status of the mutants, StuI digested CIp10 was integrated at the *RPS1* locus of all mutants. Reconstituted strains were generated by integration of the full-length open reading frame together with 1 kb of the promoter and 500 bp of terminator sequence into the SacI and NotI sites of the CIp10 plasmid. All strains were verified by Southern analysis and qRT-PCR.

### Alcian Blue binding assay

Alcian Blue assays were performed as described previously [34]. Briefly, an aliquot of 1×10^6^ stationary phase cells was washed with PBS and resuspended in 30 µg/ml Alcian Blue and incubated at room temperature for 10 min. Samples were centrifuged and 200 µl aliquots of the supernatant used to determine the concentration of unbound dye, with reference to a standard curve. The amount of Alcian Blue bound to the cell wall of *C. albicans* cells was then calculated to give an estimate of the amount of cell wall phosphomannan. Data were analysed by a one-way ANOVA, followed by post-hoc analysis including Tukey test with a 95% confidence level.

### Cell wall integrity assays

Strains were grown for 16 h in YPD media, 30°C, 200 rpm and diluted to an OD_600_ of 0.5 and 3 µl of 1∶10 serial dilutions spotted onto YPD agar and incubated at 30, 37 and 42°C for 48 h, or grown on YPD supplemented with either 100 µg/ml CFW, 0.03% SDS, 100 µg/ml Congo Red and 1 M NaCl and incubated at 30°C for 48 h.

### Activation of the cell wall integrity pathway

Activation of the cell wall integrity pathway MAP kinase Mkc1 was assessed using an phospho-p44/42 MAPK (Thr202/Tyr204) antibody (Cell Signalling Technology). Strains were grown for 16 h in YPD at 30°C, 200 rpm and diluted 1∶100 in fresh YPD medium and grown for 4 h at 30°C. As a control for Mkc1 activation, CAI-4+CIp10 was exposed to 0.032 µg/ml caspofungin for 10 min. Cells were harvested by centrifugation (3500 rpm, 5 min) and resuspended in 250 µl RE buffer (50 mM HEPES pH 7.5, 150 mM NaCl, 5 mM EDTA, 1% Triton X-100), supplemented with Roche complete proteinase cocktail. Samples were lysed with a bead beater (6×6.0 for 30 s) with 4 min on ice between each cycle. Lysates were cleared by centrifugation (13,000 rpm, 5 min) and protein concentration estimated by Bradford assay with a BSA standard curve. Proteins (15 µg) were separated by SDS-PAGE on 4–12% NuPAGE Bis-Tris gels and NuPAGE MOPS-SDS running buffer (Invitrogen) according to the manufacturer's recommendations. Proteins were transferred onto Invitrolon PVDF membranes in NuPAGE transfer buffer at 30 V for 2 h and the membrane blocked with 5% BSA in PBS-T for 1 h. Mkc1 phosphorylation was detected with a 1∶2000 dilution of phosphoP44/P42 (Erk1/2) (Thr-202/Tyr-204) rabbit monoclonal antibody (New England Biolabs, Hitchin, Hertfordshire, UK) in PBS-T with 5% BSA. Membranes were incubated overnight at 4°C. For detection, an anti-rabbit IgG-HRP conjugated antibody was used at a 1∶2000 dilution in PBS-T, 5% BSA for 1 h at room temperature. To detect Act1 levels, membrane were stripped, blocked in 10% milk in PBS-T and re-probed with an anti-Act1 antibody (Abcam, Cambridge, UK) in a 1∶2000 dilution in 5% milk in PBS-T for 2 h at room temperature. For detection an anti-mouse IgG-HRP conjugated antibody was used in a 1∶2000 dilution in PBS-T for 1 h at room temperature. Membranes were washed in PBS-T, and signals detected using an enhanced chemiluminescence (ECL) Western blotting kit (GE Healthcare) as per the manufacturer's instructions.

### HPLC analysis of the cell wall

Strains were grown in YPD, 30°C, 200 rpm for 16 h, diluted 1∶100 in fresh YPD and grown to mid-exponential phase at 37°C. Cells were washed 4 times with sterile water and lysed in a bead beater (15 cycles of 6000 rpm, 40 s with 3 min on ice between cycles). Cell pellets were washed 5 times with 1 M NaCl and pellets boiled in protein extraction buffer (50 mM Tris-HCl pH 6.8, 2% SDS, 1 mM EDTA and 0.3 M 2-mercaptoethanol) for 10 min to remove contaminating cytoplasmic proteins. Cell wall pellets were washed with water, snap-frozen in liquid nitrogen and freeze-dried. The cell wall (2–3 mg) was hydrolysed with trifluroacetic acid for 3 h at 100°C. Hydrolysed samples were washed and resuspended in water to a concentration of 10 mg/ml and 1∶10 dilutions analysed by HPLC as described previously [56] and the relative proportions of sugars in the cell wall calculated. Data were analysed by a one-way ANOVA followed by post-hoc analysis including Tukey test with a 95% confidence level using Minitab version 15.

### Fluorescence microscopy

To stain the cell wall for β-glucan, exponentially growing cells were washed and 5×10^5^ cells blocked in Buffer A (0.5% BSA, 5% HI-rabbit serum, 5 mM ETDA, 2 mM NaAzide in PBS) for 30 min. Cells were washed 3 times in Buffer B (0.5% BSA, 5 mM EDTA, 2 mM Na azide in PBS). Purified Fc Dectin-1 (kind gift from Prof G. Brown) was diluted to 3 µg/ml in Buffer A, 100 µl added to each sample which were incubated on ice for 1 h. Samples were washed 3 times in Buffer B and then incubated on ice for 45 min with 100 µl of anti-human Fc antibody conjugated to FITC diluted 1∶200 in buffer A. Samples were washed 3 times in Buffer B and fixed in 3% paraformaldehyde and cell fluorescence observed with a Zeiss Axioplan 2 microscope (63× magnification) with the Hamanatsu C4742-95 digital camera and images analysed with Openlab software v4.0.4.

To stain the cell wall for chitin and mannan, exponentially growing cells were washed in PBS and fixed in 3% paraformaldehyde for 1 h. Cells were washed in PBS and incubated in the dark with 100 µg/ml WGA-FITC to stain for chitin, or ConA-TR to stain for mannan for 45 min. Cells were washed in PBS and fluorescence observed with a Zeiss Axioplan 2 microscope (63× magnification) with the Hamanatsu C4742-95 digital camera and images analysed with Openlab software v4.0.4

### High-pressure, freeze-substitution transmission electron microscopy

*S*trains were grown in YPD, 30°C, 200 rpm for 16 h, diluted 1∶100 in fresh YPD and grown to mid-exponential phase at 37°C. To analyse the effect of the different killing methods on the cell wall architecture, cells were either heat killed at 65°C for 2 h, chemically killed by exposure to 0.05% thimerosal for 18 h, or UV killed (20 times exposure to 0.1 J/cm^2^ UV light in a UV-crosslinker). Cells were frozen in liquid nitrogen under high pressure using a Leica EM PACT2 high-pressure freezer (Leica Microsystems) and EM RTS rapid transfer system (Leica Microsystems). Freeze-substitution was carried out with a Leica EM AFS2 automatic freeze substitution system (Leica Microsystems) and EM FSP freeze substitution processor in dried acetone containing 1% OsO_4_ for 10 h. Samples were sequentially warmed to −30°C over 8 h in acetone/OsO_4_, to 20°C over 3 h in acetone, and then embedded in increasing amounts of Spurr's (epoxy) resin for 24 h. Ultra-thin sections (100 nm) were cut with a diamond knife (Diatome Ltd.) using a Leica UC6 ultramicrotome (Leica Microsystems), stained with uranyl acetate and lead citrate and imaged with a Philips CM10 transmission electron microscope (FEI). Images were recorded using Digital Micrograph software (Gatan Inc.). To ascertain the average length of the mannan fibrils for each mutant, 8 cells were randomly selected, which showed no apparent distortion from the knife, and 10 measurements made from different sections of each cell using the image software analysis program GIMP version 2.6. Data are presented as the mean and standard deviation of the 80 measurements and were analysed by a one-way ANOVA, followed by post-hoc analysis including Tukey test with a 95% confidence level using Minitab version 15.

### Mannan purification

Mannan was extracted from cells as described previously [57]–[58]. Briefly, cells were delipidated in acetone and disrupted by bead beating in deionised water, followed by autoclaving for 3 h. The cell extract was centrifuged and the supernatant split in two. To one sample, 100 mg of pronase (heated to 70°C for 30 min to remove glycosidic activity) was added and incubated at 37°C for 18 h. The other sample was untreated. The supernatant was then exposed to Fehling precipitation [57] and then dissolved in 3 M HCl. The precipitated carbohydrate and copper were removed by washing in 8∶1 methanol∶acetic acid solution until the precipitate was white to colourless. The precipitate was dried, re-suspended in water and pH adjusted to 6.5–7.0 after which the samples were lyophilised and stored at −20°C.

### Gel permeation chromatography (GPC) analysis

Duplicate mannan samples were diluted to 3 mg/ml in mobile phase and incubated at room for 15 min. The mannans were filter sterilised (0.22 µm) into sterile 1 ml injection vials. The mannans were injected at a concentration of 600 µg in 200 µl of mobile phase. GPC analyses were performed essentially as described by Kruppa and colleagues [58]. Prior to sample analysis, the GPC system was validated with water-soluble pullulan and dextran standards (1×105 to 1.66×106 g/mol). The dn/dc for mannan samples was calculated to be 0.185. Using the pullulan and dextran standards, the inter- and intra-experimental variability was <2+1%, depending upon the specific standard employed.

### NMR

Mannan (10 mg) was dissolved in ca. 600 µl D_2_O (Cambridge Isotope Laboratories, 99.8% deuterated). Proton 1D and COSY 2D NMR spectra were collected on a Bruker Avance III 600 NMR spectrometer using a CH cryoprobe operating at 333 K (60°C) in 5-mm NMR tubes. Spectral data were collected and processed as reported previously [49].

### Stimulation of peripheral blood mononuclear cells

PBMCs were isolated from the blood of healthy volunteers using Ficoll plaque as described previously [59]. *C. albicans* strains were grown in Sabouraud broth (4% glucose, 1% mycological peptone) for 16 h, 30°C, washed in PBS and resuspended to a final cell density of 5×10^6^ cells/ml in RPMI 1460 supplemented with 5% foetal calf serum and incubated at 25°C, 200 rpm for 3 h. Cells were washed in PBS and resuspended at the desired cell concentration. Yeast cells were heat killed at 65°C for 2 h and 1×10^5^ cells added to 5×10^5^ PBMCs in a final volume of 200 µl (MOI = 0.4). Cells were incubated at 37°C, 5% CO_2_ for 24 h. Samples were centrifuged (1600 rpm, 8 min) and the supernatant removed to a fresh plate. Extracellular cytokines were measured by enzyme-linked immunosorbant assay commercial kits (R&D) according to the manufacturers recommendations. Data were analysed by two-way ANOVA, followed by post-hoc analysis including Tukey test with a 95% confidence level. Minitab version 15 was used for the statistical analyses.

### *Galleria mellonella* infection model

*G. mellonella* infections were performed as described previously [60]. Briefly, fungal strains were grown to stationary phase in YPD at 30°C, 200 rpm, washed with PBS and resuspended to a final concentration of 2.5×10^7^ cells/ml. Groups of 20 *G. mellonella* larvae per strain were inoculated with 10 µl of cell suspension (2.5×10^5^ cells) by injection in the last pro-leg. Larvae were incubated at 37°C for 8 days and infection progression monitored every 12 h. Three independent biological replicates were performed and data pooled and analysed by the Kaplan-Meier Log-rank statistic and Dunnet's T-tests with a 95% confidence level using SPSS statistics version 19.

### Murine infection model

For infection experiments, *C. albicans* strains were grown in NGY medium (0.1% Neopeptone, 0.4% glucose, 0.1% yeast extract) in a rotating wheel at 30°C for 16 h. Fungal cells were harvested, washed twice in saline and then sonicated to disrupt any cell clumps. Cell suspensions were adjusted to the desired concentration by addition of saline.

Female BALB/c mice (6–8 weeks old; Harlan, UK) were randomly assigned into groups of 12 animals, with each group intravenously infected with 2×10^4^ CFU/g mouse body weight. Inoculum levels were confirmed by viable cell counts. Six mice (mice 7–12) from each group were culled on day 3, with weight change and kidney fungal burdens determined at this time to allow the infection outcome scores to be calculated [35]. The remaining six animals from each group (mice 1–6) were monitored over 28 days to determine gross virulence of the fungal strains. Mice were weighed and checked at least once daily, with any mice showing signs of severe illness and distress, or losing more than 20% of their initial body weight, being culled immediately and the kidney fungal burdens determined. Day 3 kidney fungal burdens, body weight change and infection outcome scores were compared by Mann-Whitney U test and the 28 day survival data compared by Kaplan-Meier Log-rank statistic using IBM SPSS statistics version 19.

## Supporting Information

Figure S1 **Phylogram of the** ***C. albicans MNN2*** **gene family.** A multiple sequence alignment for the six putative *MNN2* orthologues was generated using ClustalW2 (version 2.0.12) and the phylogram was created in ClustalW2.
(TIF)

Figure S2 **Deletion of** ***MNN2*** **gene family members reduces monocyte immune recognition.** Purified PBMCs were co-incubated with *C. albicans* strains at an MOI of 0.4 for 24 h. Cytokines was measured by ELISA. A) IL-1β, **B**) IL-6. Data represent the means ± SEM from 8 independent experiments, *p<0.05, **p<0.01.
(TIF)

Figure S3 **Heat killing** ***C. albicans*** **significantly alters the cell wall structure exposing underlying PAMPs.** Electron micrographs showing the ultrastructure of the cell walls of CAI-4+CIp10 after different killing methods (**A**) live, (**B**) heat killed, (**C**) UV killed and (**D**) thimerosal killed. Scale bar represents 0.5 µm.
(TIF)

Figure S4 **Thimerosal treatment significantly lowers the pro-inflammatory response.** Purified PBMCs were co-incubated with thimerosal killed *C. albicans* strains at an MOI of 0.4 for 24 h. TNFα was measured by ELISA. Data represent the means ± SD from 4 independent experiments **p<0.01.
(TIF)

Table S1 **Mannan/mannoprotein distribution in the mannosylation mutants.**
(DOCX)

Table S2 **Summary of** ***N*** **-mannan side chain composition.**
(DOCX)

Table S3 **Strains used in this study.**
(DOCX)

Table S4 **Primers used in this study.**
(DOCX)
