# Supplementary figures and images for: The Mnn2 Mannosyltransferase Family Modulates Mannoprotein Fibril Length, Immune Recognition and Virulence of Candida albicans
Source: PLoS Pathog. 2013 Apr 25;9(4):e1003276. doi: 10.1371/journal.ppat.1003276 (PMC3636026; doi:10.1371/journal.ppat.1003276)

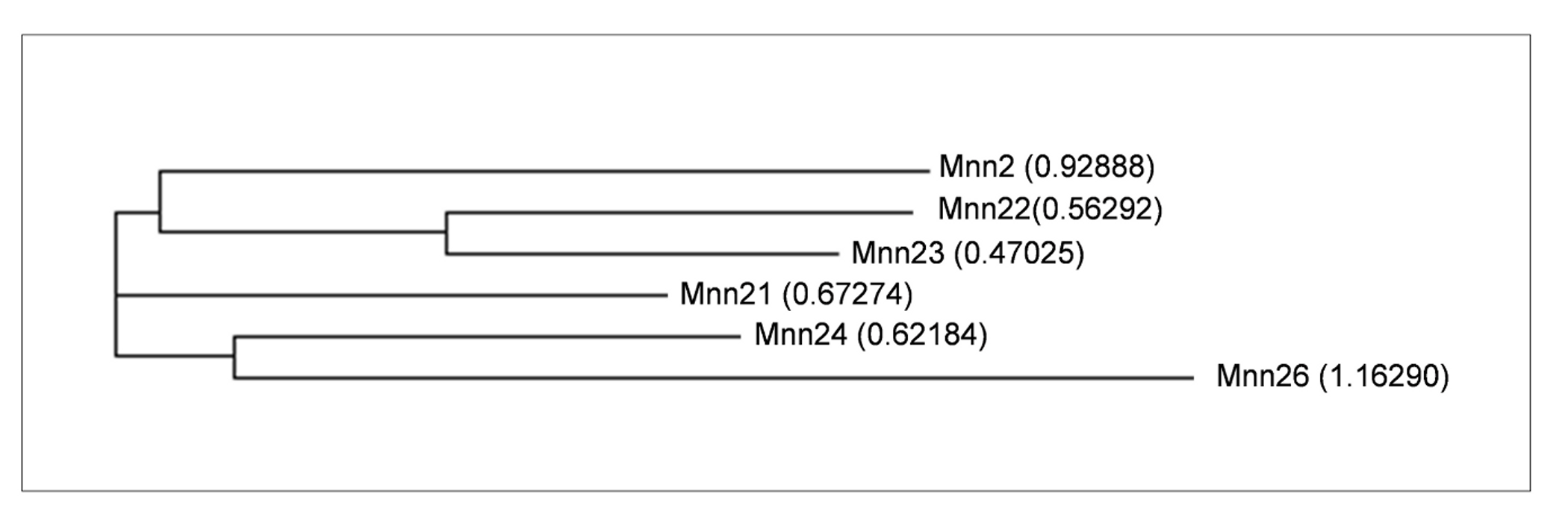

Supplement: Figure S1 — Phylogram of the C. albicans MNN2 gene family. A multiple sequence alignment for the six putative MNN2 orthologues was generated using ClustalW2 (version 2.0.12) and the phylogram was created in ClustalW2. (TIF) [file ppat.1003276.s001.tif]

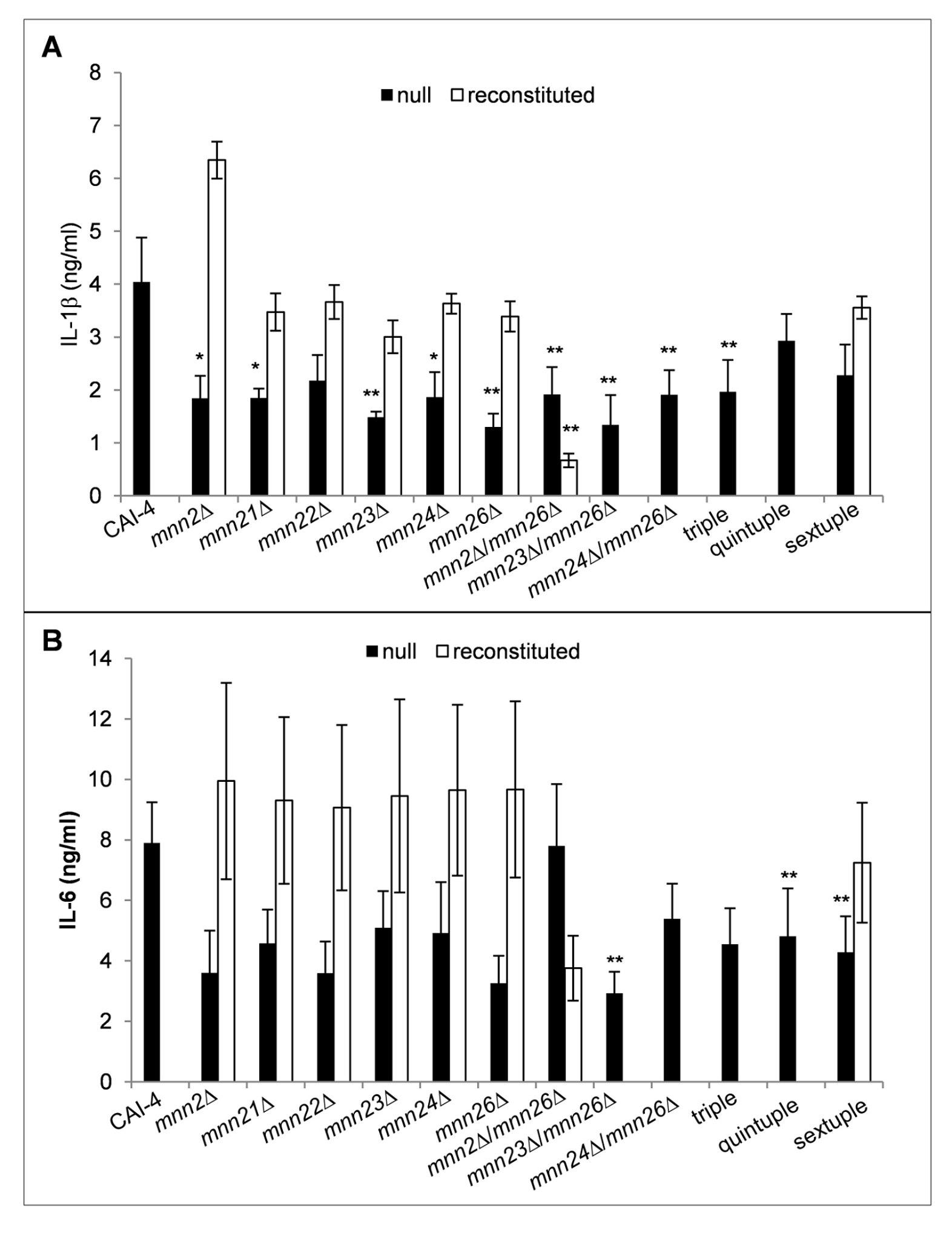

Supplement: Figure S2 — Deletion of MNN2 gene family members reduces monocyte immune recognition. Purified PBMCs were co-incubated with C. albicans strains at an MOI of 0.4 for 24 h. Cytokines was measured by ELISA. A) IL-1β, B) IL-6. Data represent the means ± SEM from 8 independent experiments, *p<0.05, **p<0.01. (TIF) [file ppat.1003276.s002.tif]

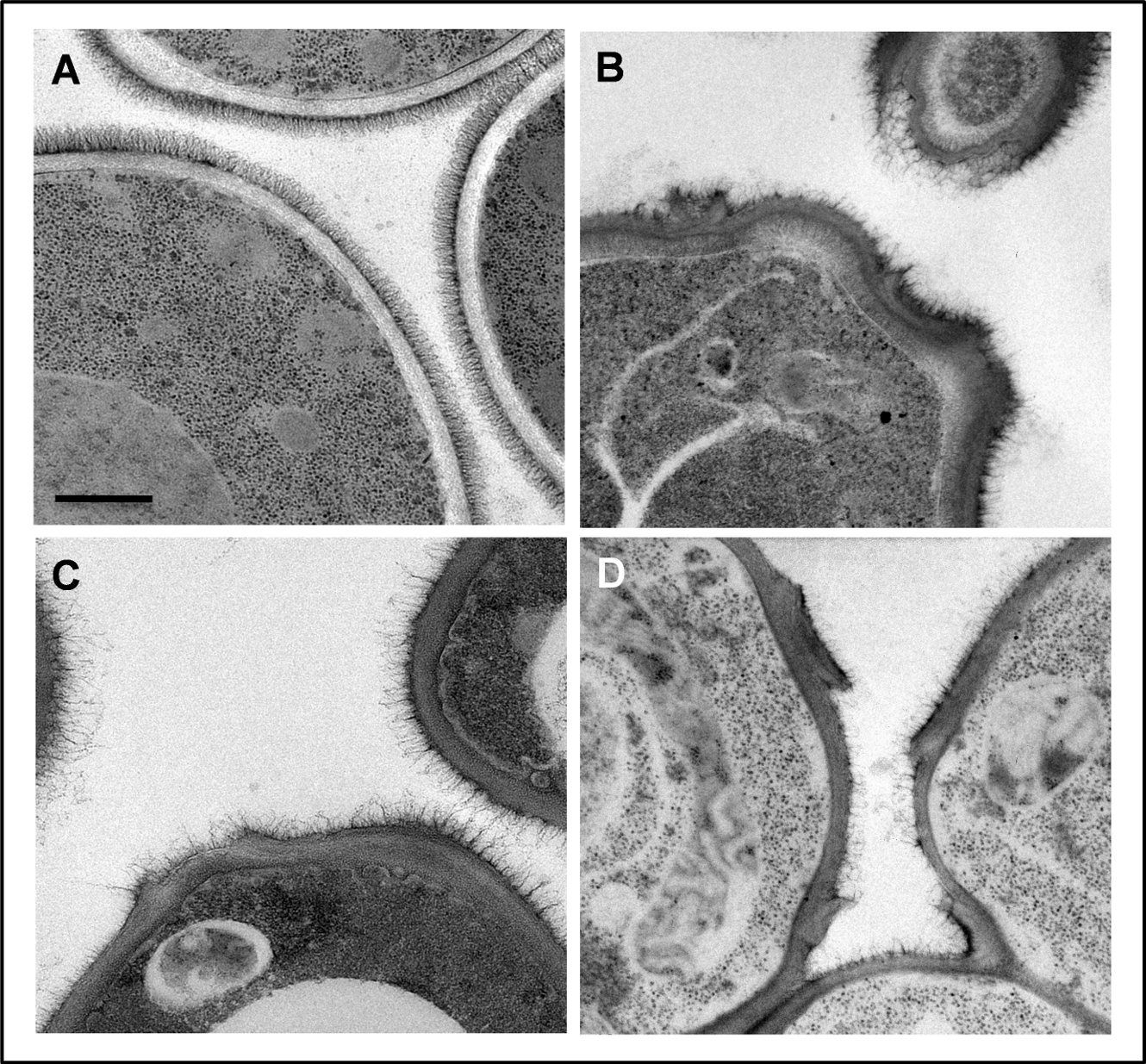

Supplement: Figure S3 — Heat killing C. albicans significantly alters the cell wall structure exposing underlying PAMPs. Electron micrographs showing the ultrastructure of the cell walls of CAI-4+CIp10 after different killing methods (A) live, (B) heat killed, (C) UV killed and (D) thimerosal killed. Scale bar represents 0.5 µm. (TIF) [file ppat.1003276.s003.tif]

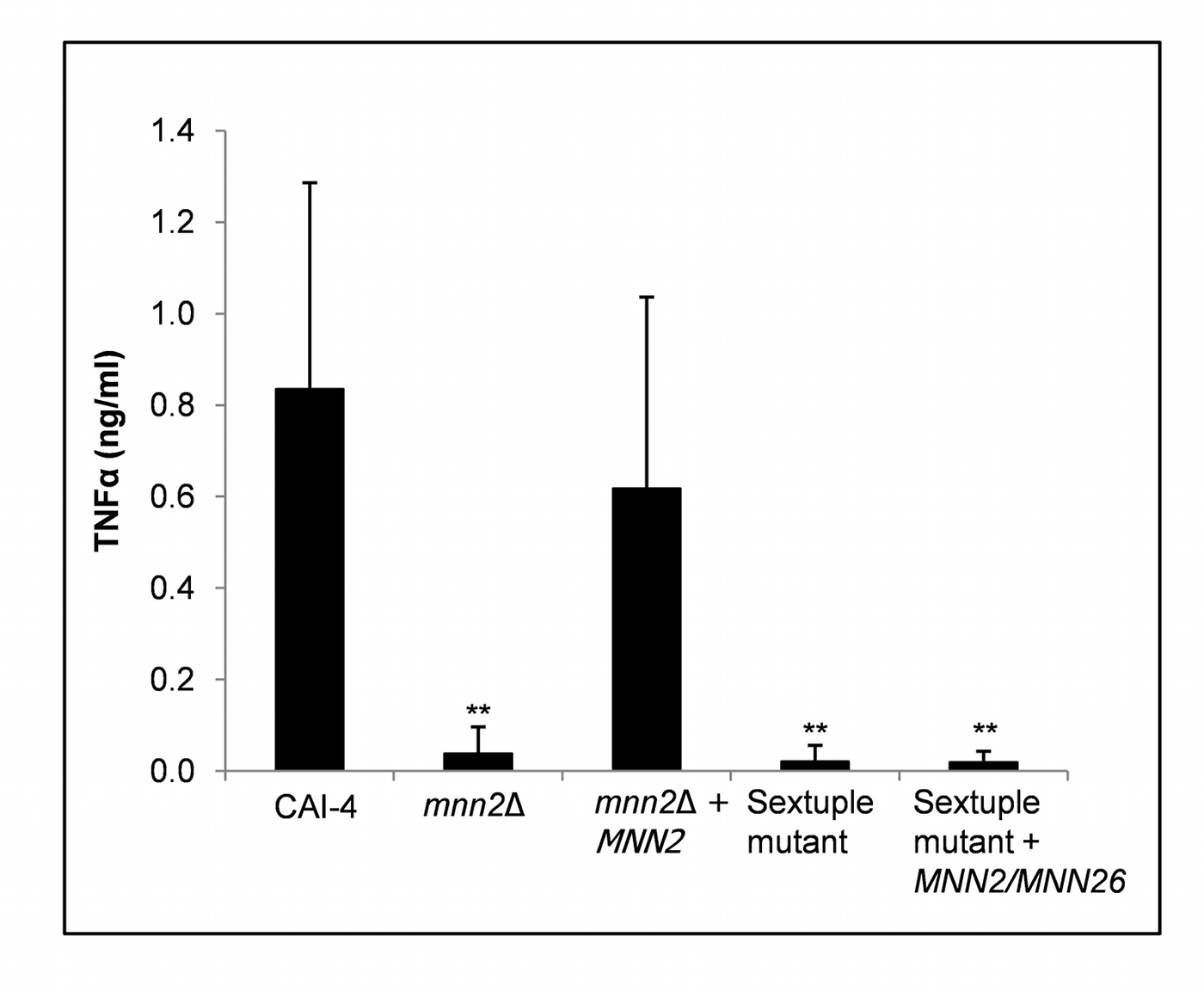

Supplement: Figure S4 — Thimerosal treatment significantly lowers the pro-inflammatory response. Purified PBMCs were co-incubated with thimerosal killed C. albicans strains at an MOI of 0.4 for 24 h. TNFα was measured by ELISA. Data represent the means ± SD from 4 independent experiments **p<0.01. (TIF) [file ppat.1003276.s004.tif]
