# Supplementary material for: The Mnn2 Mannosyltransferase Family Modulates Mannoprotein Fibril Length, Immune Recognition and Virulence of Candida albicans
Source: PLoS Pathog. 2013 Apr 25;9(4):e1003276. doi: 10.1371/journal.ppat.1003276 (PMC3636026; doi:10.1371/journal.ppat.1003276)
Supplement: Table S1 — Mannan/mannoprotein distribution in the mannosylation mutants. (DOCX) [file ppat.1003276.s005.docx]

| Sample | Mw^a^ (x10^5^ D) | %∆^b^ | Polydispersity^c^ (Mw/Mn) | %∆^b^ | Mark-Houwink^d^ (α) | %∆^b^ | Rh (nm)^e^ | %∆^b^ |
| --- | --- | --- | --- | --- | --- | --- | --- | --- |
| CAI-4 | 10.3 | --- | 5.28 | --- | 0.49 | --- | 17.5 | --- |
| *mnn26*∆ | 2.9 | -71.6 | 4.91 | 25.9 | 0.37 | -24.5 | 8.0 | -54.3 |
| *mnn2*∆/*mnn26*∆ | 0.7 | -93.2 | 1.95 | -63.1 | 0.60 | 22.4 | 5.4 | -69.1 |
| *mnn24*∆/*mnn26*∆ | 3.9 | -62.3 | 6.89 | 30.5 | 0.36 | -26.5 | 7.3 | -58.3 |
|  |  |  | Pronase | treatment |  |  |  |  |
| CAI-4 | 3.8 | -64.1 | 2.00 | -62.1 | 0.38 | -22.4 | 12.4 | -29.1 |
| *mnn26*∆ | 0.7 | -74.3 | 1.74 | -64.5 | 0.14 | -62.1 | 5.2 | -35.0 |
| *mnn2*∆/*mnn26*∆ | 0.4 | -35.7 | 1.63 | -16.4 | 0.13 | -78.3 | 4.3 | -20.4 |
| *mnn24*∆/*mnn26*∆ | 0.6 | -84.3 | 1.61 | -76.6 | 0.12 | -66.7 | 4.7 | -35.6 |

^a^Average molecular weight of the mannoprotein/mannan in Daltons

^b^Percentage change compared to CAI-4 prior to pronase treatment

^c^Polydispersity is a measure of the distribution of molecular mass in a given polymer sample and is calculated as the average molecular weight (Mw) divided by the number average molecular weight (Mn)

^d^The slope of the linear relationship between log intrinsic viscosity and log molecular mass ([Ƞ] = K_α_ M^α^) is the Mark-Houwink or α-value for a polymer system (1-3). The α value can provide insights into the solution confirmation of the polymer system

^e^Rh = hydrodynamic volume. Rh describes the volume of a polymer when it is in solution. Rh depends upon the nature of the polymer, the solvent and the temperature. In this analysis, the solvent and temperature were controlled, thus allowing examination of the polymer nature.
