# Supplementary material for: The Mnn2 Mannosyltransferase Family Modulates Mannoprotein Fibril Length, Immune Recognition and Virulence of Candida albicans
Source: PLoS Pathog. 2013 Apr 25;9(4):e1003276. doi: 10.1371/journal.ppat.1003276 (PMC3636026; doi:10.1371/journal.ppat.1003276)
Supplement: Table S2 — Summary of N -mannan side chain composition. (DOCX) [file ppat.1003276.s006.docx]

| Group | Members | Observed changes in NMR spectrum |
| --- | --- | --- |
| 1A | *mnn2*∆ + *MNN2*  *mnn26*∆ + *MNN26*  *mnn2*∆*/mnn26*∆ + *MNN2*/*MNN26* | Similar to WT |
| 1B | *mnn21*∆  *mnn21*∆ + *MNN21* | Trace amounts of mono and di- mannosyl phosphate side chains, reduced α1,3-mannose |
| 2 | *mnn2*∆  *mnn2*∆*/mnn26*∆ | Deficient in PM, reduced α1,2-mannose, shorter acid stable side chains, substituted and unsubstituted α1,6-mannose |
| 3 | *mnn26*∆  *mnn24*∆*/mnn26*∆ | Reduced PM (only long mannosyl phosphate side chains remaining),α1,3-mannose content reduced, increased unsubstituted α1,6-mannose |
| 4A | Triple mutant | Reduced PM(only long mannosyl phosphate side chains remaining), shorter acid stable side chains, increased incorporation of α1,3-mannose |
| 4B | Sextuple mutant  Sextuple mutant + *MNN2*/*MNN26* | Mainly unsubstituted α1,6-mannose |
