# Supplementary material for: The Mnn2 Mannosyltransferase Family Modulates Mannoprotein Fibril Length, Immune Recognition and Virulence of Candida albicans
Source: PLoS Pathog. 2013 Apr 25;9(4):e1003276. doi: 10.1371/journal.ppat.1003276 (PMC3636026; doi:10.1371/journal.ppat.1003276)
Supplement: Table S3 — Strains used in this study. (DOCX) [file ppat.1003276.s007.docx]

| Publication name | Name in text | Genotype | Reference |
| --- | --- | --- | --- |
| *C. albicans* Strains | |  |  |
| CAI-4 |  | *ura3∆::imm4T3h4is/ura3∆::imm434* | [1] |
| NGY152 | CAI-4 (wild type) | as CAI-4 but *RPS1/rps1∆::*CIp10 | [2] |
| NGY582 | *mnn2*∆ | *ura3∆::imm434/ura3∆::imm434* *mnn2∆::dpl200/mnn2∆::dpl200 RPS1/rps1∆::*CIp10 | This study |
| NGY584 | *mnn21*∆ | *ura3∆::imm434/ura3∆::imm434* *mnn21∆::dpl200/mnn21∆::dpl200 RPS1/rps1∆::*CIp10 | This study |
| NGY586 | *mnn22*∆ | *ura3∆::imm434/ura3∆::imm434* *mnn22∆::dpl200/mnn22∆::dpl200 RPS1/rps1∆::*CIp10 | This study |
| NGY588 | *mnn23*∆ | *ura3∆::imm434/ura3∆::imm434* *mnn23∆::dpl200/mnn23∆::dpl200 RPS1/rps1∆::*CIp10 | This study |
| NGY590 | *mnn24*∆ | *ura3∆::imm434/ura3∆::imm434* *mnn24∆::dpl200/mnn24∆::dpl200 RPS1/rps1∆::*CIp10 | This study |
| NGY592 | *mnn26*∆ | *ura3∆::imm434/ura3∆::imm434* *mnn26∆::dpl200/mnn26∆::dpl200 RPS1/rps1∆::*CIp10 | This study |
| NGY594 | *mnn2*∆/  *mnn26*∆ | *ura3∆::imm434/ura3∆::imm434* *mnn2∆::dpl200/mnn2∆:: dpl200 mnn26∆::dpl200/mnn26∆::dpl200 RPS1/rps1∆::*CIp10 | This study |
| NGY596 | *mnn23*∆/ *mnn26*∆ | *ura3∆::imm434/ura3∆::imm434* *mnn23∆::dpl200/mnn23∆::dpl200 mnn26∆::dpl200/mnn26∆::dpl200 RPS1/rps1∆::*CIp10 | This study |
| NGY597 | *mnn24*∆/ *mnn26*∆ | *ura3∆::imm434/ura3∆::imm434* *mnn24∆::dpl200/mnn24∆::His mnn26∆::Ddpl200/mnn26∆::dpl200 RPS1/rps1∆::*CIp10 | This study |
| NGY598 | Triple mutant | *ura3∆::imm434/ura3∆::imm434* *mnn2∆::dpl200/mnn2∆::dpl200 mnn22∆::dpl200/mnn22∆::dpl200 mnn23∆::dpl200/mnn23∆::dpl200 RPS1/rps1∆::*CIp10 | This study |
| NGY599 | Quintuple mutant | *ura3∆::imm434/ura3∆::imm434* *mnn2∆::dpl200/mnn2∆::dpl200 mnn22∆::dpl200/mnn2∆::dpl200 mnn23∆::dpl200/mnn23∆::dpl200 mnn24∆::dpl200/mnn24∆::dpl200*  *mnn26∆::dpl200/mnn26∆::dpl200 RPS1/rps1∆::*CIp10 | This study |
| NGY600 | Sextuple mutant | *ura3∆::imm434/ura3∆::imm434* *mnn2∆::dpl200/mnn2∆::dpl200 mnn22∆::dpl200/mnn2∆::dpl200 mnn23∆::dpl200/mnn23∆::dpl200 mnn24∆::dpl200/mnn24∆::dpl200*  *mnn26∆::dpl200/mnn26∆::dpl200 mnn21∆::dpl200/mnn21∆::dpl200 RPS1/rps1∆::*CIp10 | This study |
| NGY583 | *mnn2*∆ + *MNN2* | *ura3∆::imm434/ura3∆::imm434* *mnn2∆::dpl200/mnn2∆::dpl200 RPS1/rps1∆::*CIp10-*MNN2*) | This study |
| NGY585 | *mnn21*∆ + *MNN21* | *ura3∆::imm434/ura3∆::imm434* *mnn21∆::dpl200/mnn21∆::dpl200 RPS1/rps1∆::*CIp10-*MNN21*) | This study |
| NGY587 | *mnn22*∆ + *MNN22* | *ura3∆::imm434/ura3∆::imm434* *mnn22∆::dpl200/mnn22∆::dpl200 RPS1/rps1∆::*CIp10-*MNN22*) | This study |
| NGY589 | *mnn23*∆ + *MNN23* | *ura3∆::imm434/ura3∆::imm434* *mnn23∆::dpl200/mnn23∆::dpl200 RPS1/rps1∆::*CIp10-*MNN23*) | This study |
| NGY591 | *mnn24*∆ + *MNN24* | *ura3∆::imm434/ura3∆::imm434* *mnn24∆::dpl200/mnn24∆::dpl200 RPS1/rps1∆::*CIp10-*MNN24*) | This study |
| NGY593 | *mnn26*∆ + *MNN26* | *ura3∆::imm434/ura3∆::imm434* *mnn26∆::dpl200/mnn26∆::dpl200 RPS1/rps1∆::*CIp10-*MNN26*) | This study |
| NGY595 | *mnn2*∆ */mnn26*∆ + *MNN2/*  *MNN26* | *ura3∆::imm434/ura3∆::imm434* *mnn2∆::dpl200/mnn2∆:: dpl200 mnn26∆::dpl200/mnn26∆::dpl200 RPS1/rps1∆::*CIp10-*MNN2/MNN26*) | This study |
| NGY601 | Sextuple mutant + *MNN2/*  *MNN26* | *ura3∆::imm434/ura3∆::imm434* *mnn2∆::dpl200/mnn2∆::dpl200 mnn22∆::dpl200/mnn2∆::dpl200 mnn23∆::dpl200/mnn23∆::dpl200 mnn24∆::dpl200/mnn24∆::dpl200*  *mnn26∆::dpl200/mnn26∆::dpl200 mnn21∆::dpl200/mnn21∆::dpl200 RPS1/rps1∆::*CIp10-*MNN2/MNN26*) | This study |
| Plasmids |  |  |  |
|  | CIp10 | Candida integration plasmid | [3] |
|  | CIp10_*MNN2* | CIp10 with *MNN2* ORF with 1kb Promoter and 500 bp terminator sequence ligated as a SacI/NotI fragment | This Study |
|  | CIp10_*MNN21* | CIp10 with *MNN21* ORF with 1kb Promoter and 500 bp terminator sequence ligated as a SacI/NotI fragment | This Study |
|  | CIp10_*MNN22* | CIp10 with *MNN22* ORF with 1kb Promoter and 500 bp terminator sequence ligated as a SacI/NotI fragment | This Study |
|  | CIp10_*MNN23* | CIp10 with *MNN23* ORF with 1kb Promoter and 500 bp terminator sequence ligated as a SacI/NotI fragment | This Study |
|  | CIp10_*MNN24* | CIp10 with *MNN24* ORF with 1kb Promoter and 500 bp terminator sequence ligated as a SacI/NotI fragment | This Study |
|  | CIp10_*MNN26* | CIp10 with *MNN26* ORF with 1kb Promoter and 500 bp terminator sequence ligated as a SacI/NotI fragment | This Study |
|  | CIp10_*MNN2/MNN26* | CIp10 with *MNN2* ORF with 1kb Promoter and 500 bp terminator sequence ligated as a SacI/NotI fragment and with *MNN26* ORF with 1kb Promoter and 500 bp terminator sequence ligated as an XhoI/HindIII fragment. | This Study |

1. Fonzi WA, Irwin MY (1993) Isogenic Strain Construction and Gene Mapping in *Candida albicans*. Genetics 134: 717-728.

2. Brand A, MacCallum DM, Brown AJP, Gow NAR, Odds FC (2004) Ectopic Expression of URA3 Can Influence the Virulence Phenotypes and Proteome of Candida albicans but Can Be Overcome by Targeted Reintegration of URA3 at the RPS10 Locus. Eukaryot Cell 3: 900-909.

3. Murad AMA, Lee PR, Broadbent ID, Barelle CJ, Brown AJP (2000) CIp10, an efficient and convenient integrating vector for *Candida albicans*. Yeast 16: 325-327.
