# Supplementary material for: The Mnn2 Mannosyltransferase Family Modulates Mannoprotein Fibril Length, Immune Recognition and Virulence of Candida albicans
Source: PLoS Pathog. 2013 Apr 25;9(4):e1003276. doi: 10.1371/journal.ppat.1003276 (PMC3636026; doi:10.1371/journal.ppat.1003276)
Supplement: Table S4 — Primers used in this study. (DOCX) [file ppat.1003276.s008.docx]

| Name | Sequence |
| --- | --- |
| 19.2347-F | CCAGGCAAGGTTAGAATAATATCATTAACCACCGGCGATCCTTTCAAAGAAGCATCAAAAAGTTTTCAGActatagggcgaattggggagctc |
| 19.2347-R | GTACATCCTATTTACAAGTCCTTAAAAAATTGAAAAACTTGAAACAACTCACACCAATGTTTGTACTCTgtcgacggtatcgataagcttca |
| 19.1011-F | CGACATTATCGAATAGCCCACATTAGTGTTCGCAACTGATTTTCATACCTCCTTCCAATCCACAAACAAATCctatagggcgaattggggagctc |
| 19.1011-R | GCAACAACACATTCTCAAAACTGGATAATAATATCGCCAATGCTTTATATTGATAACCTTTGAATTCAAAgtcgacggtatcgataagcttca |
| 19.3803-F | GGAATGTCCTCCTTTCCATTTTTTCTTTTTCTATCTTTTTTTTAATTTTTTTGGAGTAATCTTTAGGTAACCctatagggcgaattggggagctc |
| 19.3803-R | CGGCAAAAACACAACAAAAATACTAGGGCAACTTGCTAGATTTCAGGCTGCTGATTAAATGGAGATTTGgtcgacggtatcgataagcttca |
| 19.4874-F | CCCGTCCCTTTCAGTTCCATTAGCCATCTTTTCATTGATATACATAATATAGAGATATTCCCACActatagggcgaattggggagctc |
| 19.4874-R | CGATTATAACGAGTTTCAATATTATTATATTATATGGACATATTTCTATTTATATGGTATCTTTTATAgtcgacggtatcgataagcttca |
| 19.1995-F | CTTATTCACTTGTTCATTCGTTCATTCGTTTACCAATACCTAATTCTGAGGGAGCTTTTTTTTTTTACCAAGActatagggcgaattggggagctc |
| 19.1995-R | GCAAAAAAACAAAAAACAAAAAACATCCAAGCAATCAATCAATACTATAATTCCAATATGACTAAACAAATgtcgacggtatcgataagcttca |
| 19.6692-F | CCTCAGTCCAAATAATTTATTTTCTTTCACCCCCCAAAAAAGATCTACAAACAAACGAACACCCTACAAGCCACGctatagggcgaattggggagctc |
| 19.6692-R | CTATCACCTTCACCATAAGCACCTTGATAAAGTAAATCAATATAAAAATCTTTGCCATTGATATTATAATgtcgacggtatcgataagcttca |
| RAH42 | GGAGAGgagctcCATCTAAACTCAATCCTAGGTCTTTC |
| RAH43 | GGAGAGgcggccgcAAACGCGGCTTTTGGACTAT |
| RAH44 | GGAGAGgagctcTAATGTAAGCTAGCACTGAGATGAGC |
| RAH45 | GGAGAGgcggccgcCATCAATTATCCCTCATGGGAA |
| RAH46 | GGAGAGgagctcGCTGTAAAGTTCGTAAATTACAAGTC |
| RAH47 | GGAGAGgcggccgcCATTTCAAAATCCTCTGGGC |
| RAH48 | GGAGAGgagctcTCAGTATCAAGTCTTTAACCTTTGAG |
| RAH49 | GGAGAGgcggccgcTCACCCACATGTAACCACTACAAC |
| RAH50 | GGAGAGgagctcGTAAGAAAGGAGTTGAGAAACAACCA |
| RAH51 | GGAGAGgcggccgcTCATCACTGTTTTGGGTTCG |
| RAH52 | GGAGAGaagcttATGATTGACTCAATCAATGCGATAG |
| RAH53 | GGAGAGctcgagCTCCAGTCCATTGTATGGGAAA |

Upper case specifies regions of homology to *C. albicans* genome

Lower case specifies regions of homology to pDDB57

Underlined lower case designates enzyme restriction sites
